# Statistical determination of synergy based on Bliss definition of drugs independence

**DOI:** 10.1371/journal.pone.0224137

**Published:** 2019-11-25

**Authors:** Eugene Demidenko, Todd W. Miller

**Affiliations:** 1 Biomedical Data Science, Geisel School of Medicine at Dartmouth, Hanover, New Hampshire, United States of America; 2 Molecular & Systems Biology, Geisel School of Medicine at Dartmouth, Hanover, New Hampshire, United States of America; University of Miami, UNITED STATES

## Abstract

Although synergy is a pillar of modern pharmacology, toxicology, and medicine, there is no consensus on its definition despite its nearly one hundred-year history. Moreover, methods for statistical determination of synergy that account for variation of response to treatment are underdeveloped and if exist are reduced to the traditional *t*-test, but do not comply with the normal distribution assumption. We offer statistical models for estimation of synergy using an established definition of Bliss drugs’ independence. Although Bliss definition is well-known, it remains a theoretical concept and has never been applied for statistical determination of synergy with various forms of treatment outcome. We rigorously and consistently extend the Bliss definition to detect statistically significant synergy under various designs: (1) *in vitro*, when the outcome of a cell culture experiment with replicates is the proportion of surviving cells for a single dose or multiple doses, (2) dose-response methodology, (3) *in vivo* studies in organisms, when the outcome is a longitudinal measurement such as tumor volume, and (4) clinical studies, when the outcome of treatment is measured by survival. For each design, we developed a specific statistical model and demonstrated how to test for independence, synergy, and antagonism, and compute the associated *p*-value.

## Introduction

The definition of synergy is one of the most controversial concepts in biology and medicine. Despite its fundamental importance in pharmacology, toxicology and experimental medicine, including cancer research, many, sometimes contradictory, definitions of synergy and drugs interaction exist in the literature [1], [2].

There are two main competing definitions of drugs independence: based on (a) Loewe [3] definition of additivity (isobologram approach) and implied combination index (CI) and (b) Bliss definition of independence [4] or, equivalently, Webb [5] fractional product. Herein, we discuss the advantages and limitations of these two major definitions, and then apply Bliss definition of independence for determination of statistically significant synergy in popular experimental and clinical trial settings in biology and medicine. Although several statistical packages in R for determination of synergy exist, such as hbim [6], mixlow [7], COMBIA [8], and CImbinator [9], they lack rigorous statistical testing and computation of the *p*-value for various treatments/drugs interaction designs under one methodological umbrella, as we propose in the present work.

### Advantages and limitations of Loewe additivity and combination index

According to Loewe, in the case of no interaction between drugs *A* and *B*, the drug combination with doses *d*_*A*_ and *d*_*B*_ leading to the same mortality *M* must satisfy the so called “median-effect” equation

$$\begin{array}{c} \frac{d_{A}}{D_{1A}}+\frac{d_{B}}{D_{1B}}=1, \end{array}$$ (1)

where *D*_1*A*_ and *D*_1*B*_ are the doses that lead to mortality *M* when applied individually. Consequently, if the left-hand side, termed Combination Index (CI), is less than one, we claim synergy (lower doses yield the same mortality *M*) and otherwise antagonism. Typically, *M* = 0.5 is used, then $D_{1A}=EC_{50}^{A}$ and $D_{1B}=EC_{50}^{B}$ are half maximum dose concentrations, and we arrive at the Loewe additivity condition on drugs independence

$$\begin{array}{c} \frac{d_{A}}{EC_{50}^{A}}+\frac{d_{B}}{EC_{50}^{B}}=1. \end{array}$$ (2)

This equations defines a segment on the plane with dose concentrations (*d*_*A*_, *d*_*B*_) splitting the positive quadrant into two parts corresponding to synergy and antagonism, and as such is visually attractive. The CI can be computed using the popular *CalcuSyn* software available at http://www.biosoft.com/w/calcusyn.htm [10] using the following steps: (a) conduct *n* experiments with drugs *A* and *B* alone at a set of doses {*d*_*Ai*_, *i* = 1, …, *n*} and {*d*_*Bi*_, *i* = 1, …, *n*}, and the same doses in combination when the drugs are given simultaneously {(*d*_*Ai*_, *d*_*Bi*_), *i* = 1, …, *n*}, (b) construct individual dose-response curves for each drug, (c) for each mortality value *M* with the drug combination find equivalent single-agent doses *D*_1*A*_ and *D*_1*B*_ by inverting the individual dose-response relationships from the previous step, and finally (d) compute *n* values of the left-hand side of Eq (1) and report the mean and standard deviation of the CI.

Although visually attractive, this approach has several limitations:

1. Statistical testing of drugs independence based on CI or Loewe definition (2) is troublesome because the uncertainty estimation of *D*_1*A*_ and *D*_1*B*_ is not taken into account. Moreover, the suggested grading of synergy based on the CI value, as suggested in [11], ignores the uncertainty of the CI estimation. A more comprehensive and scientifically accepted way to test for drug interaction is to use methods of statistical hypothesis testing through computation of the *p*- or *d*-values [12], [13]. Since these statistics are reported, synergy may be claimed when the drugs interaction is not statistically different from being independent. In short, CI <1 is not sufficient to claim synergy due to inevitable stochastic nature of experimental data.
2. The criterion for independence (1) requires knowledge of single-drug dose-response relationships, and particularly in the case of (2) knowledge of *EC*_50_. This requirement is often not satisfied when basic preliminary cell culture experiments are performed using just a few drug concentrations. Simply put, individual single-drug dose-response relationships are often not available or derived just using few data points and as such prone to large uncertainty not taken into account when computing the CI.
3. Why is the killing benchmark set at 50%? Although *EC*_50_ is an established parameter in toxicology it may be not appropriate in other applications where the point of interest is the tail of the dose-response curve. For example, epidemiology is concerned with pollutant concentration with relatively small mortality effect. On the other hand, cancer researchers mostly look at the response to the drug where cancer cell mortality is close to 1. Unlike Loewe additivity, Bliss independence does not use *EC*_50_ as the benchmark of the drug effect and therefore applicable on the entire range of doses.
4. It is well-known that the response to treatment is better to model on the log scale. Indeed, the authors of the CI approach use the log-transformed data for the single-agent dose-response relationships [14], [15], [16], [17], [18], yet drugs in Eq (1) are expressed on the linear scale. The Loewe additivity model assumes that drugs interplay in the linear fashion and as such has neither theoretical nor empirical foundation. We agree with Geary [19] who noted that “This metric is ad hoc based neither on physiology nor on formal axioms.”
5. CI is difficult to generalize to many other drug interaction settings such as cell culture in the presence of an affected control group, longitudinal organism experiments such as tumor growth in animals, or survival in clinical studies.

Most authors who discuss drug interaction provide definitions, but few publications address the topic of statistical determination of drug interaction. Data on response to treatment derived from experiments/trials in biology and medicine are subject to considerable variation. Simply reporting a CI is insufficient to claim synergy because CI < 1 may happen due to natural biological variation and stochastic nature of experiment outcomes. We assert that determination of synergy is incomplete without rigorous statistical testing against the null hypothesis of drugs independence and reporting the respective *p*-value.

The basis for our statistical developments is Bliss definition of drugs/treatments independence. We adjust this definition to various designs in biology and medicine and show how to test for synergy with maximum statistical power.

### Probabilistic interpretation of Bliss independence

The goal of this section is to illustrate Bliss independence by the formula for the probability of survival in independent events where the proportion of survived cells in assay is treated as the probability of surviving of a single cell.

According to Webb [5], drugs *A* and *B* act independently if the surviving fraction (SF) of cells or organisms upon simultaneous administration, denoted *S*_*AB*_, is equal the product of SFs, *S*_*A*_ and *S*_*B*_, when drugs are given separately, or in mathematical language,

$$\begin{array}{c} S_{AB}=S_{A}S_{B}. \end{array}$$ (3)

We say that there is synergy if *S*_*AB*_ < *S*_*A*_*S*_*B*_ and antagonism if *S*_*AB*_ > *S*_*A*_*S*_*B*_. This definition of independence has a clear interpretation: after treatment with drug *A* the proportion of cells in the population is *S*_*A*_. When drug *B* is subsequently given after drug *A*, drug *B* affects only living cells; therefore if drugs act independently, the proportion of living cells upon simultaneous drug administration is *S*_*A*_*S*_*B*_.

This definition of synergy has straightforward probabilistic interpretation [20]: associate surviving fractions *S*_*A*_ and *S*_*B*_ in the population of cells with probabilities of death of a single cell due to single-agent administration of either drug *A* or *B*, respectively. If the events of survival are denoted as *A* and *B*, then their probabilities are Pr(*A*) = *S*_*A*_ and Pr(*B*) = *S*_*B*_. If the drugs act independently, the probability of survival (as follows from the probability theory, [21]) upon simultaneous administration of both drugs is the product of individual probabilities:

$$\begin{array}{c} S_{AB}=\mathrm{Pr}\left(A\cap B\right)=\mathrm{Pr}\left(A\right)\mathrm{Pr}\left(B\right)=S_{A}S_{B}. \end{array}$$

**Fig 1:**
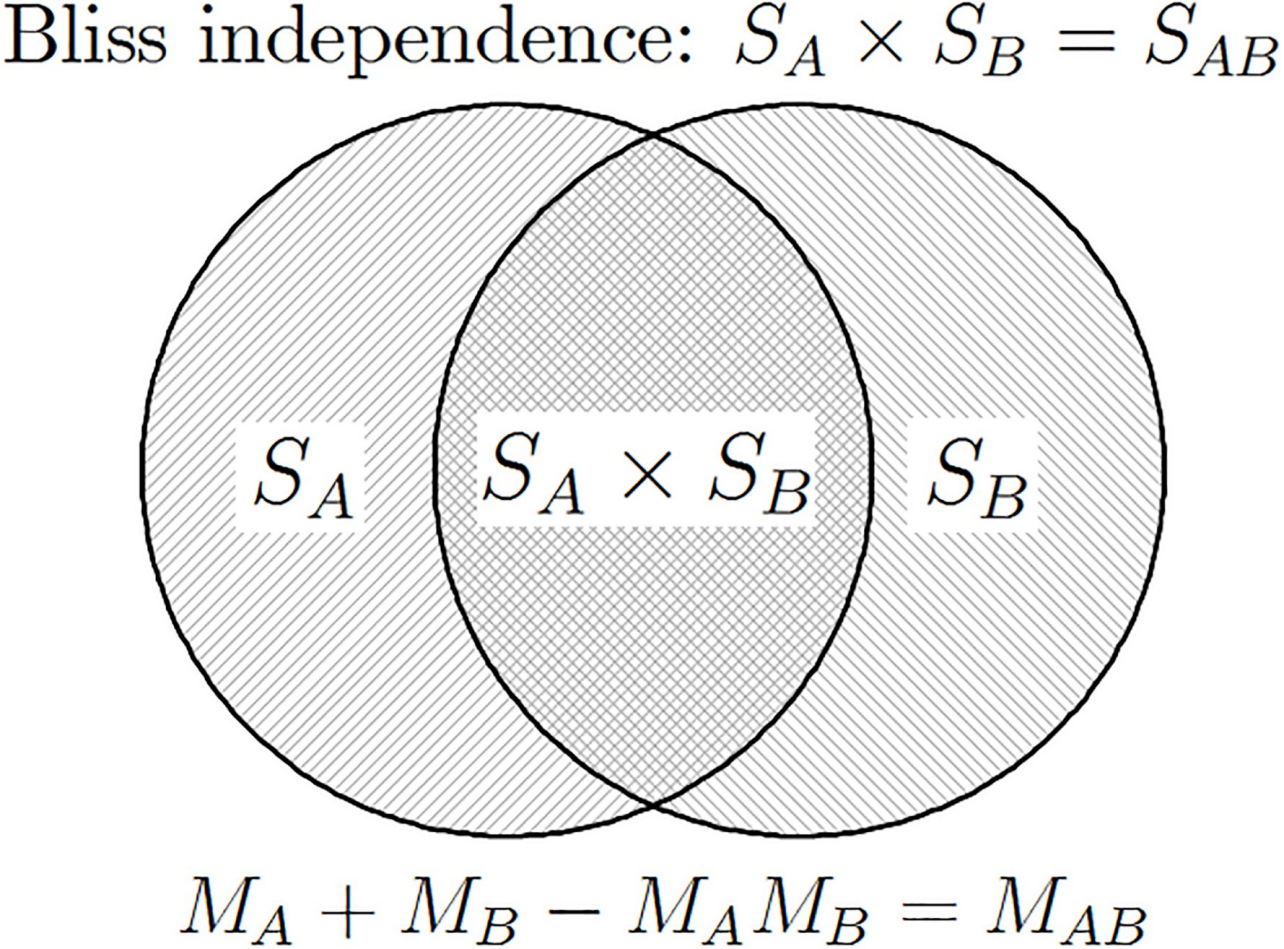
Geometrical illustration of drug independence according to Bliss (*M* = mortality, *S* = survival, *M* = 1 − *S*). The two circles depict surviving fractions in the population of cells upon single-agent administration of either drug *A* or drug *B*, denoted *S*_*A*_ and *S*_*B*_, respectively. When administered in combination, drug *B* only affects cells that survived drug *A* (and vice versa). Therefore, if drugs act independently, the surviving fraction of cells following treatment with the drug combination is the product: *S*_*A*_ × *S*_*B*_.

The independent action of drugs is illustrated in Fig 1 using the Venn diagram.

The definition of independence (3) expressed in terms of survival is equivalent to Bliss [4] independence expressed in terms of mortality,

$$\begin{array}{c} M_{A}+M_{B}-M_{A}M_{B}=M_{AB}, \end{array}$$ (4)

where *M* is the proportion of cells killed, *M* = 1 − *S*. Indeed, expressing the surviving fraction through cell kill in our definition of drugs independence we obtain

$$\begin{array}{c} S_{A}S_{B}=\left(1-M_{A}\right)\left(1-M_{B}\right)=1-M_{A}-M_{B}+M_{A}M_{B}=1-M_{AB}, \end{array}$$

which is equivalent to *M*_*A*_ + *M*_*B*_ − *M*_*A*_*M*_*B*_ = *M*_*AB*_.

The definition of drugs independence expressed in Eq (4) can be explained as follows: the sum *M*_*A*_ + *M*_*B*_ is the the total number of cells killed when two drugs act independently, but it contains double-counted cells *M*_*A*_*M*_*B*_ that must be subtracted, as cell can only die once.

Below, we systematically apply the definition of drugs independence either in the form of survival (3) or equivalently in the form of mortality (4) to various settings of drug interaction *in vitro* and *in vivo* for statistical determination of synergy or antagonism.

## Treatment interaction with single dose

This section deals with a simple yet fundamental study design for investigation of treatment (e.g. drug) interaction at specific dose. As above, the effect of a drug is associated with a surviving fraction (SF) of cells or organisms. It is critical to recognize the importance of replicates in each drug group to account for natural variation of response to treatment to make a statistically-informed statement about possible treatment independence, synergy, or antagonism. In many previous synergy analyses, such as those implemented in *CompuSyn* software mentioned above, replicates were reduced to averages by which the statistical variation of the treatment effect has been down-played. In contrast, our statistical approach to synergy is more realistic because it addresses the inevitable variation of response to treatment in cells or organisms, both and between treatment groups [20].

We underscore the importance of the single-dose assay as a preliminary step to further employment of a full-range dose investigation because it requires fewer samples. More fundamentally, synergy may exists for a specific pair of treatment doses and not an entire range of doses, therefore, a single-dose synergy may be overlooked in the traditional whole-curve dose-response approach. We emphasize that CI cannot be used with single-dose experiments because *EC*_50_ is not available.

### Treatment interactions with replicates

Let there be *n*_1_ replicate experiments in drug group *A*, *n*_2_ replicates in drug group *B*, and *n*_3_ replicates in drug group *D* when drugs with the single dose from groups *A* and *B* are given simultaneously. Since the observed SFs are positive it is convenient to model the variation of SF on the log scale, which can be expressed through the exponential function as follows:

$$\begin{array}{c} A_{j}=e^{\mu_{1}+\varepsilon_{1j}},\;B_{j}=e^{\mu_{2}+\varepsilon_{2j}},\;D_{j}=e^{\mu_{3}+\varepsilon_{3j}}, \end{array}$$ (5)

where *ε*_1*j*_, *ε*_2*j*_, *ε*_3*j*_ denote normally distributed unobserved errors with zero mean and common variance *σ*^2^. The statistical advantage of the multiplicative error scheme compared to the traditional additive scheme is two-fold: (1) the log transformation leads to the distribution that complies with the normal distribution assumption, (2) it simplifies statistical testing of synergy by turning the product of SFs into the sum.

The true unknown *μ*s are expected to be negative and connected to unknown true surviving fractions from the previous section as $S_{A}=e^{\mu_{1}}$, $S_{B}=e^{\mu_{2}}$ and $S_{AB}=e^{\mu_{3}}.$ According to Bliss, drugs act independently if

$$\begin{array}{c} \ln S_{A}+\ln S_{B}-\ln S_{AB}=0. \end{array}$$ (6)

We will test this hypothesis by statistical means using replicates in each group.

An advantage of representation (5) is that after log transformation, the model turns into an analysis of variances (ANOVA) model. Specifically, let the log fractions in each group be *y*_1*i*_ = ln *A*_*i*_, *y*_2*i*_ = ln *B*_*i*_ and *y*_3*i*_ = ln *D*_*i*_. Then the system (5) can be rewritten as an ANOVA model [13], [22], [23] with three groups,

$$\begin{array}{c} y_{ij}=\mu_{i}+\varepsilon_{ij},\;i=1,2,3,\;j=1,...,n_{i} \end{array}$$

where *n*_*i*_ is the number of replicates in the *i*th group. Now the drugs independence can be conventionally expressed in terms of *μ*s as the linear null hypothesis

$$\begin{array}{c} H_{0}:\mu_{1}+\mu_{2}-\mu_{3}=0. \end{array}$$

This hypothesis is tested using the test statistic *T* which has a *t*-distribution with $\sum_{i=1}^{3}n_{i}-3$ degrees of freedom, namely,

$$\begin{array}{c} T=\frac{\left({\bar{y}}_{1}+{\bar{y}}_{2}-{\bar{y}}_{3}\right)\sqrt{\sum_{i=1}^{3}n_{i}-3}}{\sqrt{\sum_{i=1}^{3}\sum_{j=1}^{n_{i}}{\left(y_{ij}-{\bar{y}}_{i}\right)}^{2}\sum_{i=1}^{3}n_{i}^{-1}}}, \end{array}$$ (7)

where ${\bar{y}}_{1},$ ${\bar{y}}_{2}$ and ${\bar{y}}_{3}$ are the average log fractions in groups *A*, *B* and *D* [13]. To test the null hypothesis of drugs independence, we compute the two-sided the *p*-value; to test for synergy or antagonism we compute the one-sided *p*-value. We emphasize that the *t*-test (7) is exact and it does not rely on the large-sample theory as several other tests have been used for testing synergy, including [24], [25]. Exact tests are especially important in biomedical research because the sample size is usually small. In order to make our statistical test tractable, we assume the equal-variance assumption across groups, common assumption in statistical theory [26].

Other than log transformation can be applied to surviving fraction to comply with the normal distribution assumption. For example, one may use the logit transformation, ln (*S*/(1 − *S*)), or inverse Weibull cumulative distribution function transformation [27]. The advantage of the log transformation is that the Bliss definition of independence turns into a linear equation easy to test by standard linear statistical theory.

Several authors used ANOVA models to test Loewe additivity condition based on the CI, or test the Bliss independence null hypothesis *H*_0_: *S*_*AB*_ − *S*_*A*_*S*_*B*_ = 0 using the traditional *t*-test. Particularly, the CI-based ANOVA models have been criticized in [2] and [28]. We have to comment that the normal distribution assumption is not adequate here because it makes the possibility of CI or surviving fraction taking negative values, which makes no sense. We, however, test the Bliss independence on the log scale, as follows from Eq (6), and therefore normal distribution and negative values are permissible.

#### Example: Daphnia acute test with CuSO_4_ and NiCl

**Fig 2:**
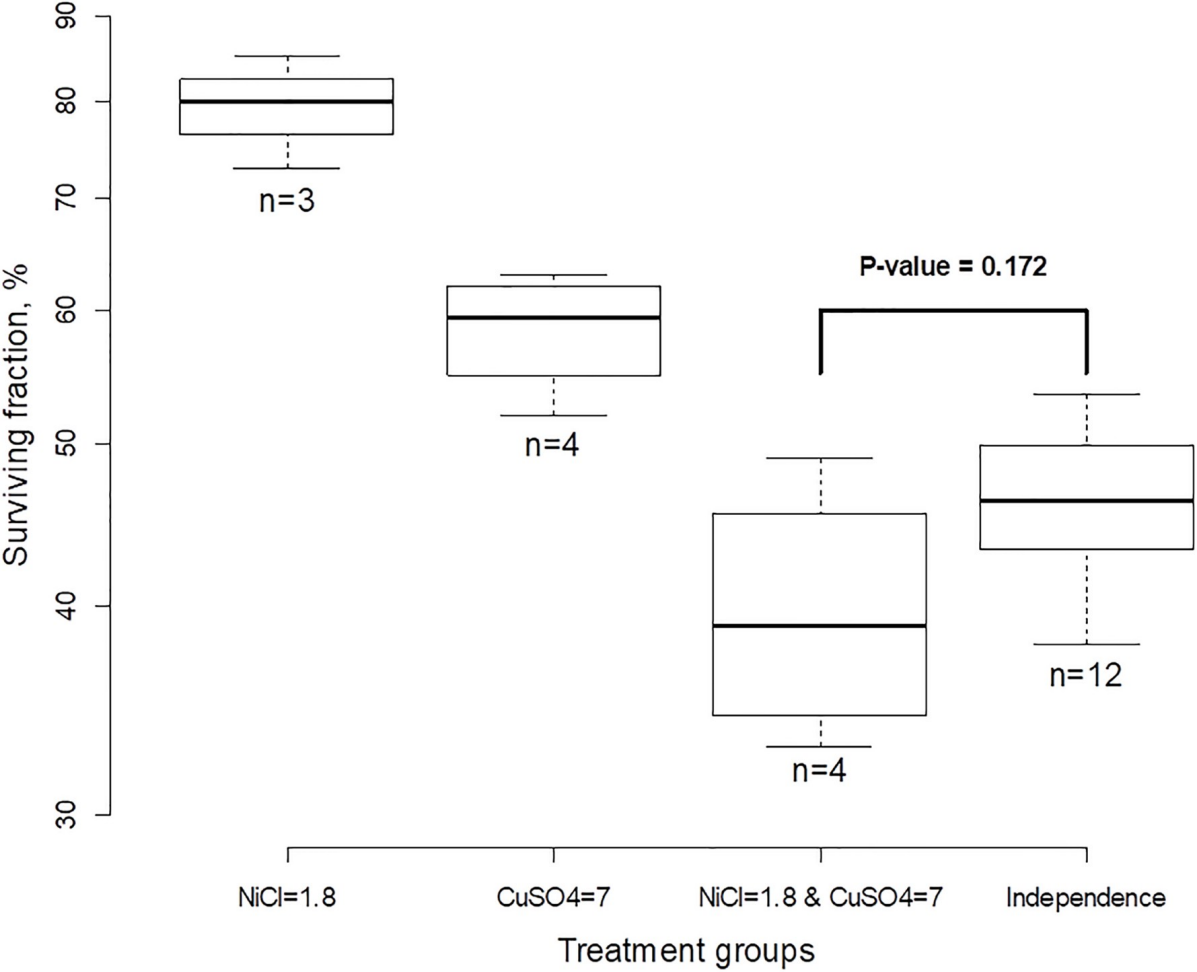
Daphnia acute tests with single-dose stressors NiCl and CuSO_4_. Although surviving fraction when the two stressors are applied simultaneously is smaller than if they were acting independently, the “detected” synergy of 17% is not statistically significant.

We illustrate treatment interaction with a single dose of each treatment using 48-hour acute tests with *Daphnia* [29], [30]. In each experiment, organisms in water were treated with two stressors, a single dose of NiCl (1.8 mg/L), CuSO_4_ (7.0 *μ*g/L) or the combination, and the numbers of surviving organisms were counted after 48 hours. There were three repeated experiments (replicates) with NiCl, four replicates with CuSO_4_ and four replicates with the combination. The results are portrayed in Fig 2. The right-most group “Independence” was derived from the individual groups by computing SF under the assumption that treatments act independently, i.e. on the log scale as pairwise sums *y*_1*j*_ + *y*_2*k*_, where *j* = 1, 2, 3 and *k* = 1, 2, 3, 4. Note that although all calculations with SF were done on the log scale we used the original scale in % on the *y*-axis for easy interpretation (the scale is not uniform). For these data, ${\bar{y}}_{1}+{\bar{y}}_{2}-{\bar{y}}_{3}=0.156$ and the ratio of SF under independence to SF when both treatments are applied simultaneously is exp(*y*_1_ + *y*_2_ − *y*_3_) = 1.168 which means that synergy was 17%, which means that 17% more cells would survive if stressors acted independently. However, according to the *t*-test (7) this result is not statistically significant because *p* = 0.172—there is no sufficient evidence to reject the null hypothesis that NiCl and CuSO_4_ act independently.

It is worthwhile to note that determination of statistical significance of drug interaction is impossible using the isobologram and Combination Index (CI) approaches because (a) replicates are substituted with average values so that variation between replicates is ignored and (b) the *EC*_50_ values are not available because the data exist only for single dose.

### Treatment interaction in the presence of control group

In many biological experiments treatments, elicit delayed but not immediate effects on cell/organism. During such time, cells may die or proliferate by natural means, which can be captured through inclusion of a control/sham group *C* and the associated surviving fraction *S*_*C*_. Following our probabilistic approach we equate the proportion of cells that survived in group *C* with the probability of dying of a single cell, *S*_*C*_ = Pr(*C*). Now we turn our attention to the group treated with drug *A*. Appealing to probability theory, we treat fraction *S*_*A*_ as the proportion of cells killed by drug *A* but exclude those cells that naturally died as in the control group, *S*_*A*_ = Pr(*A* ∩ *C*). Thus, the complimentary probability 1 − *S*_*A*_ is the probability of death either due to drug *A* or natural causes. The probability of survival associated with the effect of drug *A* can be viewed as the conditional probability [21]

$$\begin{array}{c} \mathrm{Pr}\left(A|C\right)=\frac{\mathrm{Pr}(A\cap C)}{\mathrm{Pr}(C)}=\frac{S_{A}}{S_{C}}. \end{array}$$

Note that this formula connects the treatment effect of drug *A* with the observed surviving fraction in groups *A* and *C*. We do not observe the conditional event *A*|*C*, but treat this as imaginary occurrence to account for cell loss due to natural death, or reversely, to cell proliferation observed in control group. A similar probability of survival can be obtained for the population of cells treated with drug *B* alone, and for simultaneous treatment with both drugs *A* and *B*,

$$\begin{array}{c} \mathrm{Pr}\left(B|C\right)=\frac{\mathrm{Pr}(B\cap C)}{\mathrm{Pr}(C)}=\frac{S_{B}}{S_{C}},\;\mathrm{Pr}\left(AB|C\right)=\frac{\mathrm{Pr}(AB\cap C)}{\mathrm{Pr}(C)}=\frac{S_{AB}}{S_{C}}. \end{array}$$

Following the rule of independence defined in Eq (3) we arrive at a more general definition of synergy when the population in the control group reduces due to natural cell death,

$$\begin{array}{c} \frac{S_{A}}{S_{C}}\times \frac{S_{B}}{S_{C}}>\frac{S_{AB}}{S_{C}}. \end{array}$$

or equivalently *S*_*A*_*S*_*B*_ > *S*_*C*_*S*_*AB*_. When the population in the control group does not change we arrive at the definition of synergy as expressed in Eq (3). The condition of drugs independence on the log scale turns into the additive equation,

$$\begin{array}{c} \ln S_{A}+\ln S_{B}-\ln S_{C}-\ln S_{AB}=0, \end{array}$$ (8)

similar to the previous case without an affected control group given by Eq (6). We say that drugs are synergistic if ln *S*_*A*_ + ln *S*_*B*_ − ln *S*_*C*_ − ln *S*_*AB*_ > 0.

The statistical model for testing hypothesis (8) is similar to what was described earlier with the addition of replicates from the control group $C_{j}=e^{\mu_{0}+\varepsilon_{0j}}.$ Denoting log SF as *y*_0*i*_ = ln *C*_*i*_ the system can be rewritten as an ANOVA model with four groups as follows:

$$\begin{array}{c} y_{ij}=\mu_{i}+\varepsilon_{ij},\;i=0,1,2,3,\;j=1,...,n_{i} \end{array}$$

where *μ*_0_ = ln *S*_*C*_, *μ*_1_ = ln *S*_*A*_, *μ*_2_ = ln *S*_*B*_, *μ*_3_ = ln *S*_*AB*_. Now the drugs independence assumption (8) can be expressed as a null hypothesis:

$$\begin{array}{c} H_{0}:\mu_{1}+\mu_{2}-\mu_{3}-\mu_{0}=0. \end{array}$$ (9)

Under this hypothesis the ratio of the mean difference to the standard error

$$\begin{array}{c} T=\frac{\left({\bar{y}}_{1}+{\bar{y}}_{2}-{\bar{y}}_{3}-{\bar{y}}_{0}\right)\sqrt{\sum_{i=0}^{3}n_{i}-4}}{\sqrt{\sum_{i=0}^{3}\sum_{j=1}^{n_{i}}{\left(y_{ij}-{\bar{y}}_{i}\right)}^{2}\sum_{i=0}^{3}n_{i}^{-1}}}. \end{array}$$ (10)

is distributed according to the *t*-distribution with $\sum_{i=0}^{3}n_{i}-4$ degrees of freedom. We claim a statistically significant synergy if *T* is positive and exceeds the critical value of the *t*-distribution [13].

Slinker [31] defines synergy in terms close to (9) and urges use of ANOVA to test the independence of treatment interaction. However, Slinker was not specific regarding what biological quantity/effect to use for *μ* and simply referred to “experimental outcome.” Instead, (a) we apply treatment interaction to the data when the outcome of an experiment is survival fraction, (b) we derived the null hypothesis using the conditional probability, and (c) as a part of our formal derivation we use the log scale i.e. *μ* is the log SF and therefore comply with the normal distribution assumption.

#### Example. Testing for drug synergy for cancer cells

**Fig 3:**
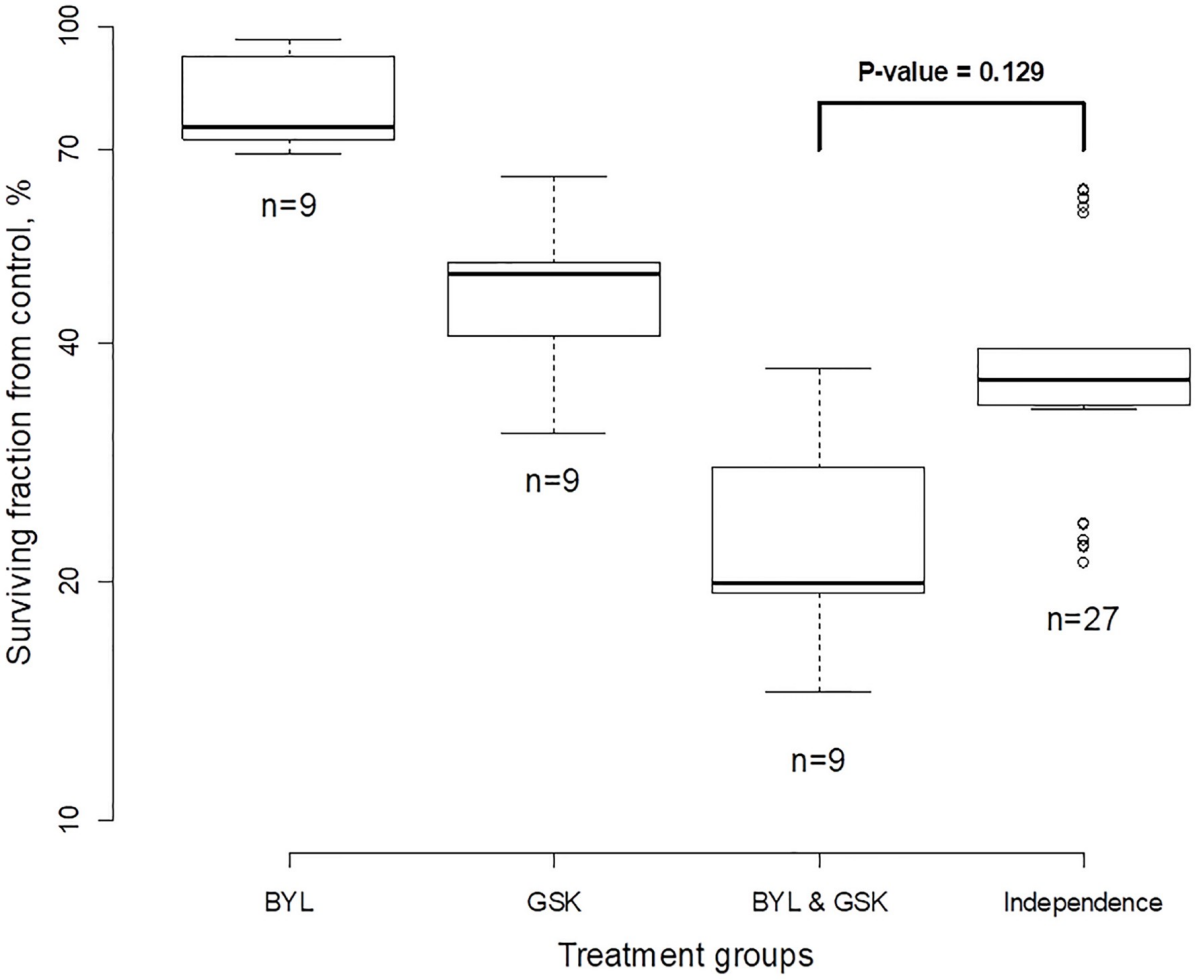
Drug interaction for testing synergy of BYL and GSK at single doses in breast cancer cells.

These cell culture data come in the form of the surviving fraction of cells per dish. These experiments were intended to test for synergy between the drugs BYL and GSK in ZR75-1 breast cancer cells *in vitro* [32]. There were four treatment groups and three replicates per group: control, 1*μM* BYL, 1*μM* GSK, and the combination at 1*μM* each. For each treatment group, we first computed 9 pairwise differences from the control group on the log scale as ln *A*_*j*_ − ln *C*_*k*_, ln *B*_*j*_ − ln *C*_*k*_, and ln *D*_*j*_ − ln *C*_*k*_; see Fig 3. Secondly, data were plotted with the *y*-axis as % surviving cells with respect to the control group. The analysis and graphics representation are similar to the *Daphnia* case with no control group. The right-most box depicts the imaginable surviving fraction (SF) if drugs acted independently according to Bliss. Those values were computed based on 27 triple combinations from groups *A*, *B*, and *C* as follows: (ln *A*_*j*_ − ln *C*_*k*_) + (ln *B*_*l*_ − ln *C*_*k*_). If BYL and GSK acted independently the two right-most boxes would be the same height. However, the SF under the additive assumption (right-most box) is higher than that from the actual data (combination group); therefore, we may expect treatment synergy between BYL and GSK. Indeed, the ratio of SF under Bliss independence to SF from the combination group is 1.65, which can be interpreted as 65% synergy. The problem is that the SFs exhibit variation and the *T*-statistic computed by formula (10) yields 1.7 producing *p* = 0.129 that indicates that observed synergy is not statistically significant.

Thus, despite a fairly high value of synergy, the *p*-value is not below 0.05 because of the variation in the group surviving fraction.

## Dose-response relationship

In this section, we discuss statistical determination of treatment interaction when cell/organism survival experiments are conducted using a range of doses. Two types of analyses are presented: (a) model-free statistical determination, when survival experiments with the individual and combination treatments are dose-escalated in parallel, and (b) estimation of a bivariate dose-response relationship with any set of doses.

### Model-free statistical determination of synergy

This method was originally suggested by Webb [5] and is commonly referred to as the fractional product method [1]. The idea is compare the SF from the combination group with the SF computed as the product of the SFs from the two single-agent groups. Although the idea is straightforward, no rigorous statistical model or statistical test for synergy has been developed. For example, [33] suggest estimating the beta-slope coefficient in the linear regression of the SF in the combination group on the product of the SFs from single-agent treatment groups. However, this approach has a number of limitations: (i) it does not comply with the normal distribution assumption because the SF is in the range from 0 to 1, (ii) it assumes that the single-agent SFs do not have variation (fixed) but the SF from the combination group does, (iii) no statistical test for the Bliss independence hypothesis *H*_0_: *β* = 1 has been developed. In contrast, statistical methods described below comply with the normal distribution because the analysis of the SF is carried out on the log scale and rigorous statistical tests of Bliss independence are developed for different assay designs.

We propose to test Bliss independence by employing the theory of a linear model similar but not equivalent to the ANOVA model discussed above. Two types of design are studied here: incomplete and complete pairwise design. In incomplete design, the same number of doses are used in individual and combination treatments. In complete design, all pairwise combination of treatments are required. An advantage of the model-free statistical determination of synergy is that no dose-response relationship is required, but experiments with combined treatments must be included with the same concentrations as individual treatment. Although the models described below do not account for an affected control group, their generalization is straightforward following the idea in the preceding section.

#### Incomplete pairwise design

Let *S*_*Ai*_ and *S*_*Bi*_ be SFs of cells in the *i*th experiment with dose *d*_*Ai*_ of drug *A* and dose *d*_*Bi*_ of drug *B* for *i* = 1, 2, .., *n*. Let *S*_*Di*_ be the SF in the *i*th experiment when drugs *A* and *B* are combined with doses (*d*_*Ai*_, *d*_*Bi*_). If drugs are acting independently ln *S*_*Ai*_ + ln *S*_*Bi*_ and ln *S*_*Di*_ must be close. To test the difference, the following statistical model under multiplicative error scheme is suggested. Denote *x*_*i*_ = ln *S*_*Ai*_, *y*_*i*_ = ln *S*_*Bi*_, and *z*_*i*_ = ln *S*_*Di*_ and let

$$\begin{array}{c} x_{i}=\mu_{i}+\varepsilon_{i},\;y_{i}=\tau_{i}+\zeta_{i},\;z_{i}=\mu_{i}+\tau_{i}+\delta +\eta_{i},\;i=1,...,n, \end{array}$$

where *μ*_*i*_ and *τ*_*i*_ are the true SFs from groups *A* and *B* on the log scale, and *ε*_*i*_, *ζ*_*i*_, *η*_*i*_ are independent and identically normally distributed error terms with zero mean and constant variance. Parameter *δ* is of interest: if *δ* = 0, drugs act independently; if *δ* < 0, we have synergy; if *δ* > 0, we have antagonism. To test the null hypothesis *H*_0_: *δ* = 0 we take the difference *z*_*i*_ − (*x*_*i*_ + *y*_*i*_) and estimate the delta-parameter as $\bar{z}-\left(\bar{x}+\bar{y}\right),$ where the bar indicates the average. The paired *t*-test with *n* − 1 degrees of freedom is used to test the null hypothesis. Note that the traditional (unpaired) *t*-test to compare *z*_*i*_ with *x*_*i*_ + *y*_*i*_ is not appropriate here because these observations/measurements have means dependent on *i* because SF depends on the dose.

As an example, we illustrate this test using the data from [34]. For these data, the estimate of the delta-parameter is 1.64 which indicates that there is antagonism between the drugs with two-sided *p* = 6.43 × 10^−7^.

#### Complete pairwise design

Under this design simultaneous application of drugs is conducted at all pairs of the doses. Let *S*_*Ai*_ be the SF of cells in the *i*th experiment with dose *d*_*Ai*_ of drug *A* where *i* = 1, 2, …, *n*_*A*_. Similarly, let *S*_*Bj*_ be the SF in the *j*th experiment of drug *B* with dose *d*_*Bj*_, where *j* = 1, 2, …, *n*_*B*_. It is assumed that in *n*_*A*_*n*_*B*_ experiments (group *D*), drugs are given in combination (*d*_*Ai*_, *d*_*Bj*_) and result in the SF *S*_*Dij*_. According to Bliss independence we need to compare values ln *S*_*Ai*_ + ln *S*_*Bj*_ with ln *S*_*Dij*_. As in the previous model, we assume a multiplicative error scheme with *x*_*i*_ = ln *S*_*Ai*_, *y*_*j*_ = ln *S*_*Bj*_, and *z*_*ij*_ = ln *S*_*Dij*_, where

$$\begin{array}{c} x_{i}=\mu_{i}+\varepsilon_{i},\;y_{j}=\tau_{j}+\zeta_{j},\;z_{ij}=\mu_{i}+\tau_{j}+\delta +\eta_{ij},\;i=1,...,n_{A},j=1,...,n_{B}, \end{array}$$

where *μ*_*i*_ and *τ*_*j*_ are unknown and subject to estimation log SF, *δ* is the parameter of interest, and *ε*_*i*_, *ζ*_*j*_, and *η*_*ij*_ are error terms and considered as independent and identically normally distributed with zero mean and constant variance *σ*^2^. Drugs *A* and *B* are independent if *δ* = 0. The drugs are synergistic if *δ* < 0 and antagonistic if *δ* > 0. As in the previous models hypothesis testing of *δ* reduces to the *t*-distribution with the test statistic

$$\begin{array}{c} T=\frac{\bar{x}+\bar{y}-\bar{z}}{s\sqrt{\left(1/n_{A}+1/n_{B}+1/\left(n_{A}n_{B}\right)\right)}} \end{array}$$

where

$$\begin{array}{c} s^{2}=\frac{\sum_{i=1}^{n_{A}}{\left(x_{i}-\bar{x}\right)}^{2}+\sum_{j=1}^{n_{B}}{\left(y_{j}-\bar{y}\right)}^{2}+\sum_{i=1}^{n_{A}}\sum_{j=1}^{n_{B}}{\left(z_{ij}-\bar{z}\right)}^{2}}{n_{A}+n_{B}+n_{A}n_{B}-3} \end{array}$$

is the pooled variance and *n*_*A*_ + *n*_*B*_ + *n*_*A*_*n*_*B*_ − 3 is degrees of freedom.

### Choice of drug concentration

It is important to chose the right set of doses to conduct experiments when dose-response relationship is estimated or when synergy is being tested by statistical means. A simple rule for estimation of individual dose-response curves has been devised in [29] to optimally choose drug concentrations that induce mortality (or reduce SF) to 0.122 and 0.878, the pivotal points on the logistic response curve.

In choosing optimal drug concentrations to detect synergy it should be remembered that under the null hypothesis of drug interaction the SF is the product of individual SFs. The optimal statistical identification of the product (e.g., when *p*-value is minimal) is when variance is maximal. If SFs are computed from Bernoulli random variables (dead/alive) for two drugs *X* and *Y*, we want to maximize *var*(*XY*) where Pr(*X* = 1) = *S*_*A*_ and Pr(*Y* = 1) = *S*_*B*_. The optimal choice is when *S*_*A*_*S*_*B*_ = 0.5. As an example, we suggest the combinations of drugs that leads to products 0.4, 0.5, and 0.6, which can be achieved using the following individual SFs: 0.6, 0.7, and 0.77, or their combination, depending on the size of the study.

### The two-drug copula mortality function

In the analysis above, we were concerned with statistical testing for synergy. But if synergy is detected, how do we predict the mortality probability given two synergistic treatments? The answer relies on the two-drug dose-response function. Several two-drug dose-response relationships, as a part of the response surface methodology, have been suggested [1], but none satisfy the properties formulated below. Most relationships been developed in connection with CI, not Bliss independence, such as [14]; see reviews [2] and [35]. Several authors associated the interaction effect with the product of drug concentrations as customarily used in the linear model framework [36], but justification is lacking. A typical example of an *ad hoc* dose-response model [37] is a quadratic polynomial and as such can be negative and/or not an increasing function of dose concentration.

The goal of this work is to present a novel rigorous two-drug copula mortality function *M*(*d*_*A*_, *d*_*B*_) that satisfies the following properties:

1. Log scale: Model *M* is expressed on the log scale (i.e., drug concentrations enter the model as ln *d*_*A*_ and ln *d*_*B*_).
2. Singe-drug inheritance in the absence of the other drug, the two-agent copula model collapses to a single-drug model, *M*_*A*_(*d*_*A*_), or *M*(*d*_*A*_, 0) = *M*_*A*_(*d*_*A*_), the mortality function of drug *A* applied alone. Similarly, we require that *M*(0, *d*_*B*_) = *M*_*B*_(*d*_*B*_), the mortality function of drug *B*.
3. Independence/synergy parameter: the model depends on parameter *ρ*, which determines independence, synergy, or antagonism, or more rigorously, (a) when *ρ* = 0 the model collapses to the Bliss independence model *M*(*d*_*A*_, *d*_*B*_) = *M*_*A*_(*d*_*A*_) + *M*_*B*_(*d*_*B*_) − *M*_*A*_(*d*_*A*_)*M*_*B*_(*d*_*B*_), (b) when *ρ* < 0 we have synergy *M*(*d*_*A*_, *d*_*B*_) > *M*_*A*_(*d*_*A*_) + *M*_*B*_(*d*_*B*_) − *M*_*A*_(*d*_*A*_)*M*_*B*_(*d*_*B*_), and (c) when *ρ* > 0 we have antagonism, i.e. *M*(*d*_*A*_, *d*_*B*_) < *M*_*A*_(*d*_*A*_) + *M*_*B*_(*d*_*B*_) − *M*_*A*_(*d*_*A*_)*M*_*B*_(*d*_*B*_).

A two-drug model simplifies statistical determination of independence, synergy, or antagonism: estimate *ρ*; if the null hypothesis *H*_0_: *ρ* = 0 is not rejected, we claim Bliss independence; otherwise, synergy or antagonism depending on the sign of *ρ*. None of the two-agent dose-response models suggested previously satisfy the above properties. The goal of this section is to introduce a family of novel dose-response functions that describe the mortality effect in the presence of two drugs (*d*_*A*_, *d*_*B*_) for any given single-drug models *M*_*A*_(*d*_*A*_) and *M*_*B*_(*d*_*B*_), and use it for statistically determination of synergy.

The basis for our creation is (a) recognition that the mortality function with one or more drugs can be viewed as a cumulative distribution function (cdf) of one or multiple random variables, respectively, routinely used in probability theory, and (b) apply copula to construct a two-agent mortality function using individual mortality functions treated as marginal cdfs in the probability theory [38], [39], [40], [41]. As shown in Appendix (Supporting Information), the two-drug copula mortality function is expressed through a double integral over the bivariate standard normal density with the correlation coefficient *ρ* as

$$\begin{array}{c} M\left(x,y;\rho \right)=1-\frac{1}{2\pi \sqrt{1-\rho^{2}}}\int_{\Phi^{-1}\left(M_{A}\left(x\right)\right)}^{\infty }\int_{\Phi^{-1}\left(M_{B}\left(y\right)\right)}^{\infty }e^{-\frac{1}{2(1-\rho^{2})}\left(u^{2}-2\rho uv+v^{2}\right)}dudv, \end{array}$$ (11)

where Φ stands for the standard normal cumulative distribution function with Φ^−1^ its inverse and *x* = ln *d*_*A*_ and *y* = ln *d*_*B*_ are the drug concentrations on the log scale. It is shown that this integral can be expressed through a single integral. In fact, our approach defines not just a two-drug function but a family of functions with any combination of single-drug models, such as Hill, probit, or Weibull [30].

Correlation coefficient *ρ* receives a new meaning in our copula mortality function. When *ρ* < 0, drugs complement each other and therefore the killing effect increases. Conversely, when *ρ* > 0 the killing effect diminishes when drugs are applied simultaneously. The following theorem defines the limits of mortality when two drugs are completely antagonistic or synergistic as extreme cases when *ρ* approaches 1 or −1.

**Theorem. The properties of the two-drug copula mortality function**. (a) Complete antagonism: when two drugs are the same, i.e. *M*_*A*_ = *M*_*B*_ and *ρ* → 1, then *M*(*d*_*A*_, *d*_*B*_) → *M*_*A*_(max(*d*_*A*_, *d*_*B*_)). (b) Complete synergy: when *ρ* → −1 then *M*(*d*_*A*_, *d*_*B*_) → *M*_*A*_(*d*_*A*_) + *M*_*B*_(*d*_*B*_) if *M*_*A*_(*d*_*A*_) + *M*_*B*_(*d*_*B*_) < 1 and *M*(*d*_*A*_, *d*_*B*_) → 1 otherwise.

The proof is in Appendix (Supporting Information). Complete antagonism indicates that the drugs have completely overlapping effect, e.g. when the drugs affect the same set of cellular receptors, so the mortality is defined by the maximum dose. Complete synergy occurs when the affected cellular receptors do not overlap; then the joint mortality is the sum of single mortalities.

**Fig 4:**
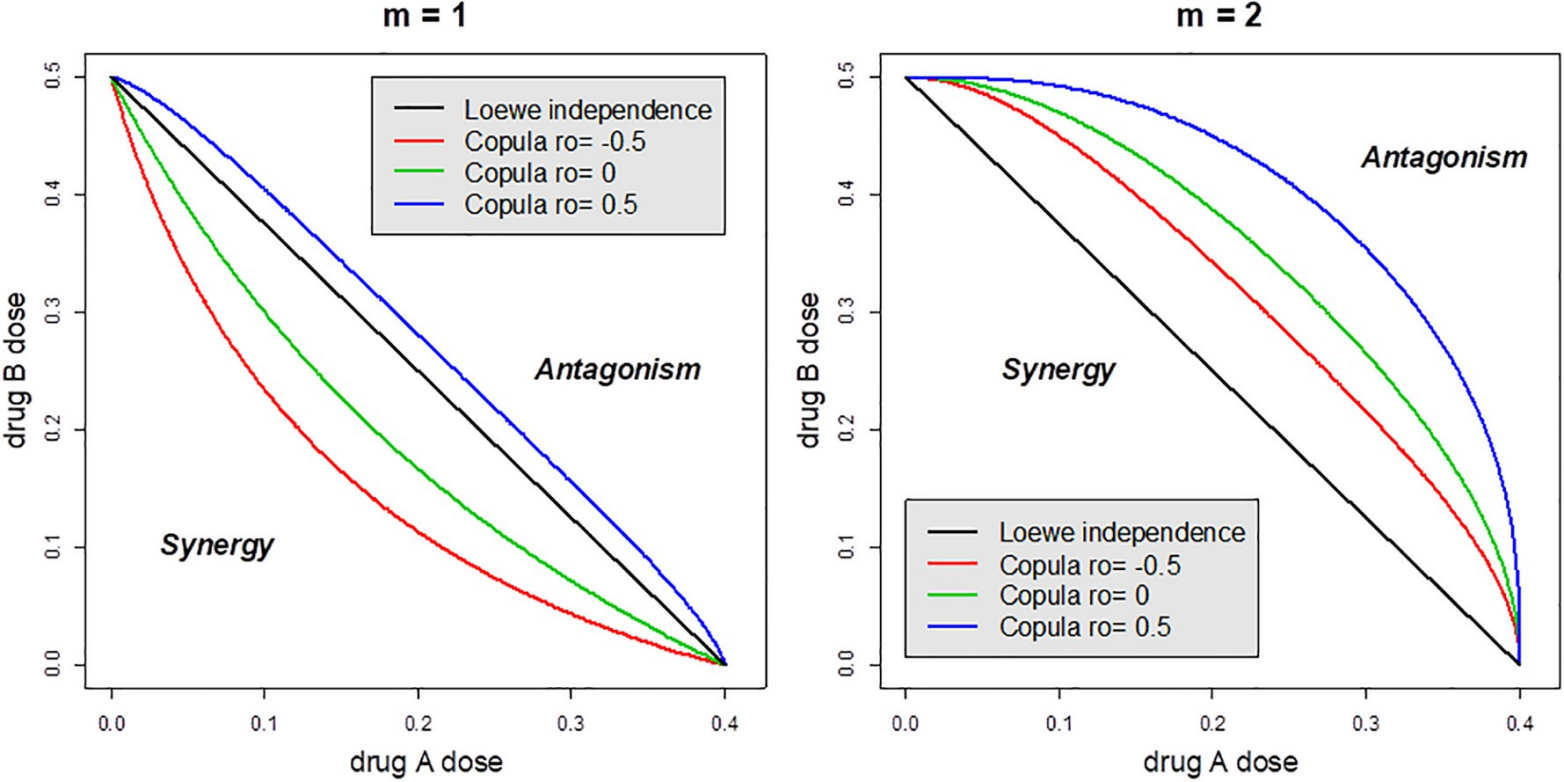
Illustration of the two-drug copula mortality function using EC_50_ contours. When drugs have moderate effect on mortality (*m* = 1) synergy will be underestimated by the Loewe approach and overestimated if drugs have a strong effect (*m* = 2).

The two-drug copula function is illustrated in Fig 4 by contours of the two-drug function (11) with mortality held at the 50% level. To comply with the Loewe additivity approach, the single-drug mortality functions for this example share the same slope *m* and therefore give rise to the two-drug function. Specifically, drugs *A* and *B* have the same *m* = 1, 2 but different $EC_{50}^{A}=0.4$ and *EC*_50_ = 0.5 and therefore the Loewe independence is depicted as a segment (black), which connects the two *EC*_50_s. The fact that Loewe and Bliss independence are not equivalent is well-known and can be seen from this plot (the black and green lines are different). When *m* = 1 synergy will be overestimated and antagonism will be underestimated using Loewe independence, compared with that derived from our two-drug copula model, because the green contour line (*ρ* = 0) is below the black Loewe segment. The bottom-left corner depicts synergy and the top-right corner depict antagonism. If drugs complement each other (*ρ* = −0.5), a smaller dose can lead to the same 50% kill, and on the other hand, increased doses are required to get 50% kill. On the other hand, if drugs have overlapping effect (*ρ* = 0.5) and are therefore antagonistic. Conclusions change when drugs have a stronger effect on mortality (*m* = 2): synergy may be claimed according to the copula model while the Loewe approach concludes antagonism (the green contour line is above the black Loewe segment).

#### Example: Testing lethal effects of insecticides

To illustrate our two-agent copula mortality model, we used classic data on lethal effects of two insecticides, rotenone and pyrethrins, on fruit flies from experiments described by Finney [42] and later analyzed by [15] for determination of synergy/antagonism. The original data contain two series of experiments with the ratio of rotenone:pyrethrins as 1:5 and 1:15 mixtures; we used only the first data series for illustration. Using the CI, the authors declared the 1:5 mixture to be “mildly synergistic.” We apply our statistical model to determine if the synergy is statistically significant.

**Fig 5:**
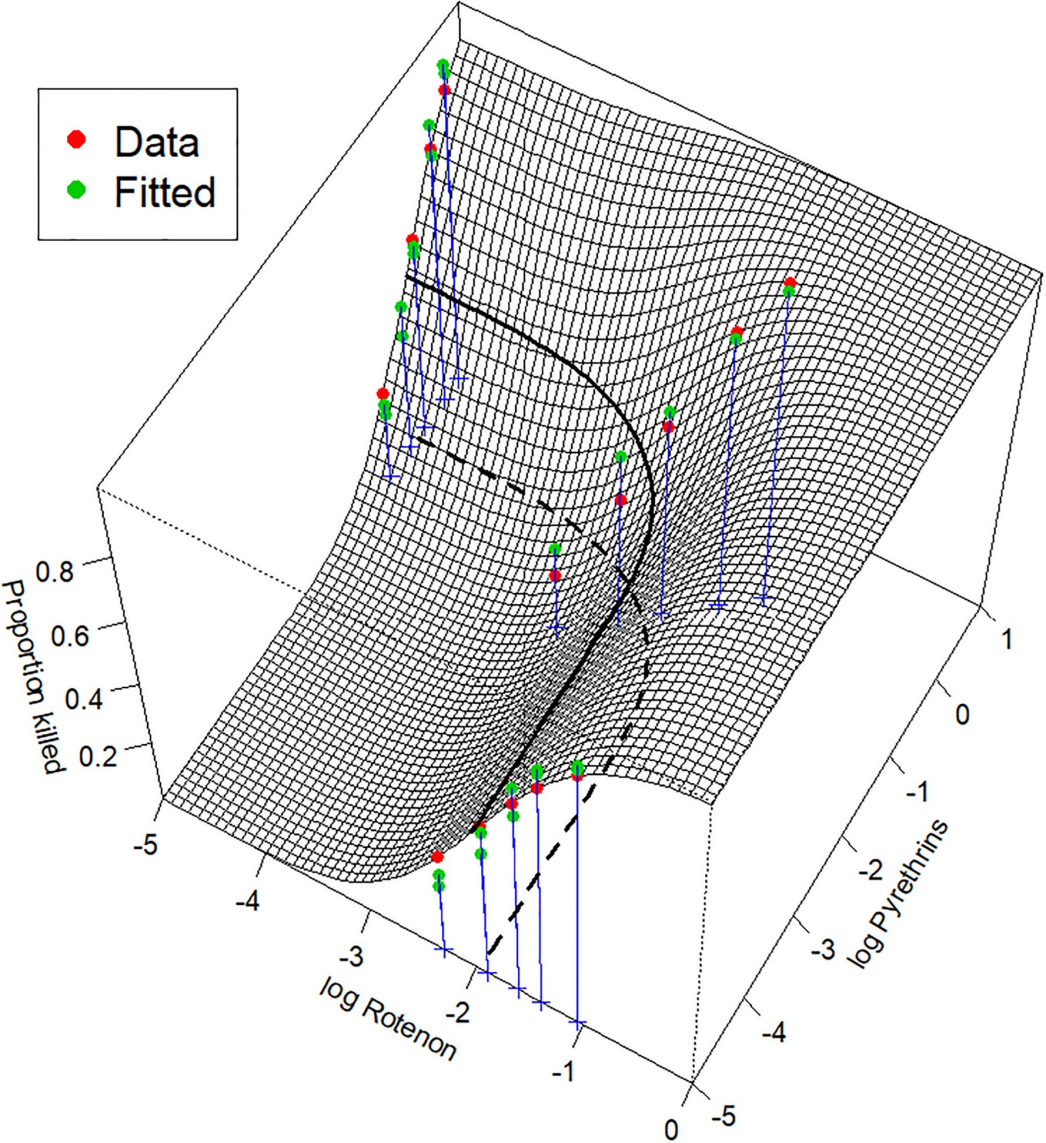
Fit of 1:5 ratio of rotenone to pyrethrins data by the probit-based two-drug copula mortality function. The bold curve depicts the combination of doses of the two drugs that leads to the expected 50% fly kill (its projection on the xy-plane is depicted as the dashed line). The synergy between drugs is statistically significant with *p*-value = 0.0086.

**Fig 6:**
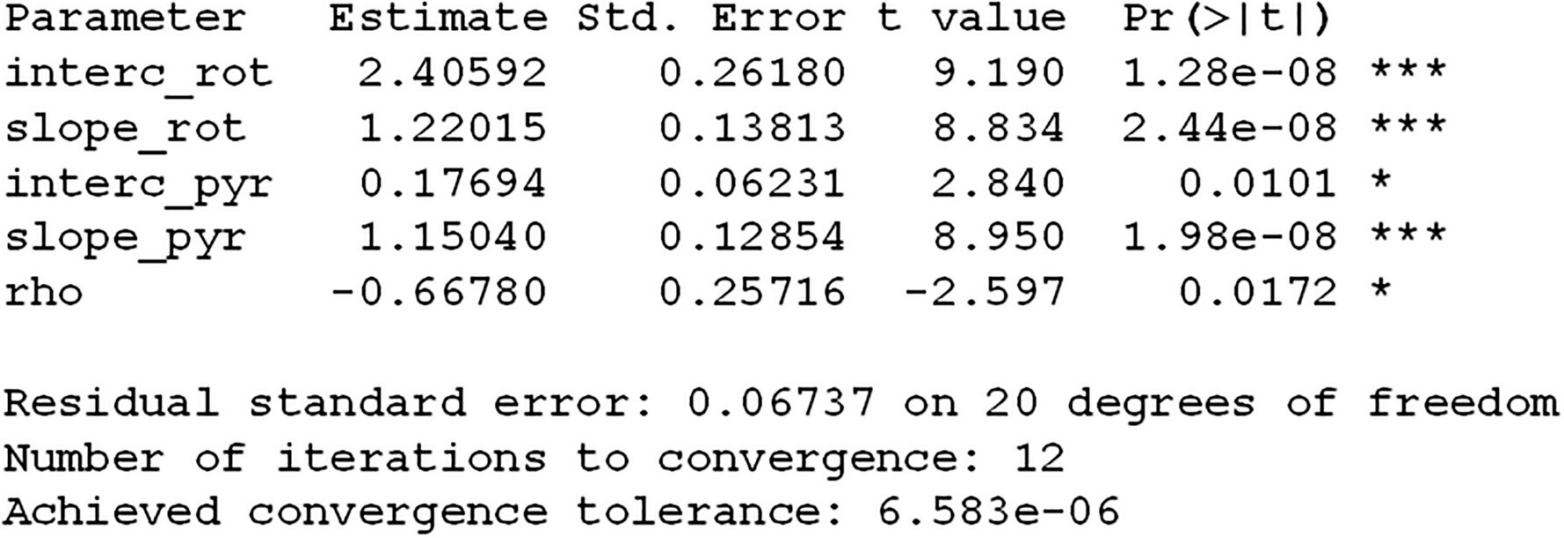
The output of program nls for estimation of the probit-based two-drug copula model (11).

The estimated probit-based two-drug copula mortality function depicted as a surface in 3D is shown in Fig 5 and the output of the nonlinear least squares algorithm (the nls function in R) is listed in Fig 6.

All parameters are statistically significant. Note that the two drugs have slopes greater than one. They termed by Chou and Talalay [15] as of “high order,” which explains why the 50% contour line points away from the origin as in the right plot in Fig 4. The rho-parameter = −0.6678, and this negative value confirms synergy. The null hypothesis that drugs act independently is rejected with *p* = 0.0172, and the one-sided *p*-value for the synergy is 0.0086 = 0.0172/2. Details on the algorithmic implementation and the R code are found in Supporting Information.

## Tumor growth experiments *in vivo*

Before moving to clinical studies, drugs are often tested in animals *in vivo*. Several authors apply CI for tumor volume at a specific time point [43], [44], [45], [46]. Here we apply Bliss definition to tumor volume data measured over time *t* in four groups of animals: control (*C*), group with drug *A*, group with drug *B*, and group *D* with a combination of drugs *A* and *B* (all drugs are given in a single dose). Our analysis of drug interaction relies on the following assumptions: (1) tumor volume is reflective of number of cancer cells, (2) at the time when treatments begins (baseline), tumor volume is the same in all four groups, (3) drugs are given over the course of treatment and result in exponential growth (or decay) of tumors with a group-specific rate. The combination of these assumptions leads to group-specific exponential growth/decay of tumors

$$\begin{array}{c} C\left(t\right)=e^{\alpha +\beta_{0}t},\;A\left(t\right)=e^{\alpha +\beta_{1}t},\;B\left(t\right)=e^{\alpha +\beta_{2}t},\;D\left(t\right)=e^{\alpha +\beta_{3}t},\;t\geq 0 \end{array}$$

where *C*, *A*, *B* and *D* are tumor volumes measured at time *t* (e.g. day or weeks), and *β*_0_, *β*_1_, *β*_2_, and *β*_3_ are the respective tumor growth rates. An important assumption is that tumors grow or shrink according to an exponential law over the course of a drug treatment regimen. Consequently, it is advantageous to plot tumor volume data on the log scale because exponential growth/decay appears as a straight line and the slope can be visually assessed [47]. The tumor growth dynamic may not be seen when drugs are given for a short period of time or with a one-time treatment. With such treatment tumor volume over time may give a U-shaped curve when a tumor shrinks, reaches nadir, and then resumes growth requiring a different types of model, namely, regrowth curves described in [20].

Below we demonstrate that the hypothesis of drug interaction in tumor growth experiments can be reduced to drug interaction in the presence of a control group as discussed above. Indeed, fix time *t* and consider the proportion of survived cells in three treatment groups as *S*_*A*_ = *A*(*t*)/*C*(*t*), *S*_*B*_ = *B*(*t*)/*C*(*t*), and *S*_*AB*_ = *D*(*t*)/*C*(*t*). Following Bliss definition, drugs act independently if *S*_*AB*_ = *S*_*A*_*S*_*B*_ or on the log scale if ln *S*_*AB*_ = ln *S*_*A*_ + ln *S*_*B*_. However,

$$\begin{array}{ccc} \ln S_{A}+\ln S_{B} & = & \ln A(t)-\ln C(t)+\ln B(t)-\ln C(t)=\ln A(t)+\ln B(t)-2\ln C(t),\\ \ln S_{AB} & = & \ln D(t)-\ln C(t) \end{array}$$

and therefore drugs independence turns into the equation *α* + *β*_1_*t* + *α* + *β*_2_ *t* − 2(*α* + *β*_0_*t*) = *α* + *β*_3_*t* − *α* − *β*_0_*t*. After cancellation of time *t*, the null hypothesis of Bliss independence can be rewritten in terms of the rate coefficients,

$$\begin{array}{c} H_{0}:\beta_{1}+\beta_{2}-\beta_{3}-\beta_{0}=0. \end{array}$$

This condition is similar to condition (9), but is expressed in terms of rate of tumor growth. We say that there is synergy if *β*_1_ + *β*_2_ − *β*_3_ − *β*_0_ > 0 is statistically supported by the *p*-value computed from the estimates of the slope and their variances obtained from the output of the R function lme from the library nlme (see details in Supporting Information). In other words, synergy means that rate of tumor growth in the drug combination group yields a slower rate than if drugs acted independently, compared to the control group. Typically, each group of animals is estimated separately using the theory of mixed models [20] to account for differences in individual trajectory and baseline tumor volume. The following example illustrates this approach.

### Example: Combination of two drugs (EHT1864 and fulvestrant) for breast tumor treatment

**Fig 7:**
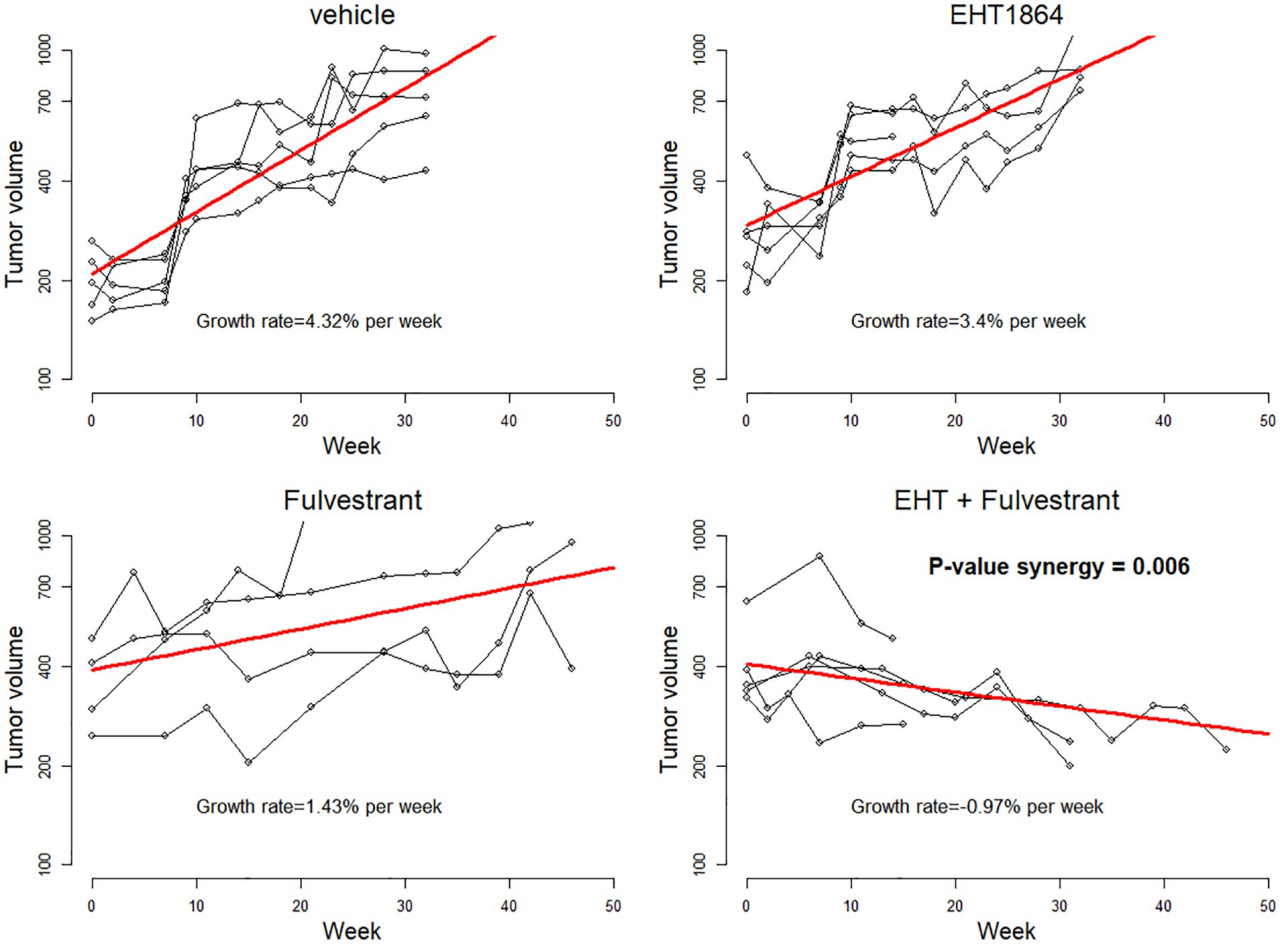
Exponential growth in four groups of mice for testing synergy between EHT1864 and fulvestrand for breast tumor treatment estimated by linear mixed model (see details in supporting information). The data are plotted on the log scale; the tickmarks correspond to the original volume in mm^3^. There is a statistically significant synergy between the drugs with *p*-value = 0.006.

The data [48] and fit are depicted in Fig 7. The R code is listed in Supporting Information. Since exponential function on the log scale turns into a linear function it is advantageous to plot tumor volume data on the log scale but show tickmarks on the *y*-axis using actual tumor volume. To address inter-animal heterogeneity the data are fitted by a linear function with random effect that reflects the differences of tumor volume at the time of treatment [20]. For example, the rate of breast tumor growth in the control group is estimated as ${\hat{\beta }}_{0}=0.0432,$ which means that tumors grow by 4.32% every week if treated with vehicle. The combination of drugs (EHT1864 plus fulvestrant) induces tumor shrinkage at the rate of 0.97% per week. The above formula for *p*-value yields 0.006, indicating a statistically significant drug interaction between EHT1864 and fulvestrant.

## Survival curves

An advantage of Bliss independence over Loewe additivity, and the related CI, is that drug interaction can be applied to clinical data when the outcome of treatment is survival, e.g. recurrence-free survival, time to recurrence/progression, progression-free survival, or overall survival. We then treat proportions of patients that survive by time *t* as the probability of an individual to stay alive.

Let treatments *A* and *B*, when applied separately, lead to survival curves *S*_*A*_(*t*) and *S*_*B*_(*t*), respectively, and let *S*_*AB*_(*t*) be the survival curve if drugs are applied simultaneously. If drugs were acting independently, as follows from Bliss definition we expect the survival curve to be

$$\begin{array}{c} 1-\left(1-S_{A}\left(t\right)\right)\left(1-S_{B}\left(t\right)\right). \end{array}$$ (12)

If drugs/treatments are synergistic, the observed survival *S*_*AB*_(*t*) must be greater than (12), or geometrically, the curve *S*_*AB*_(*t*) is above the curve under independence assumption (12). To test for drugs independence, we compare the survival curve (12) with *S*_*AB*_(*t*) using standard statistical tests for survival curves, such as the logrank test.

**Fig 8:**
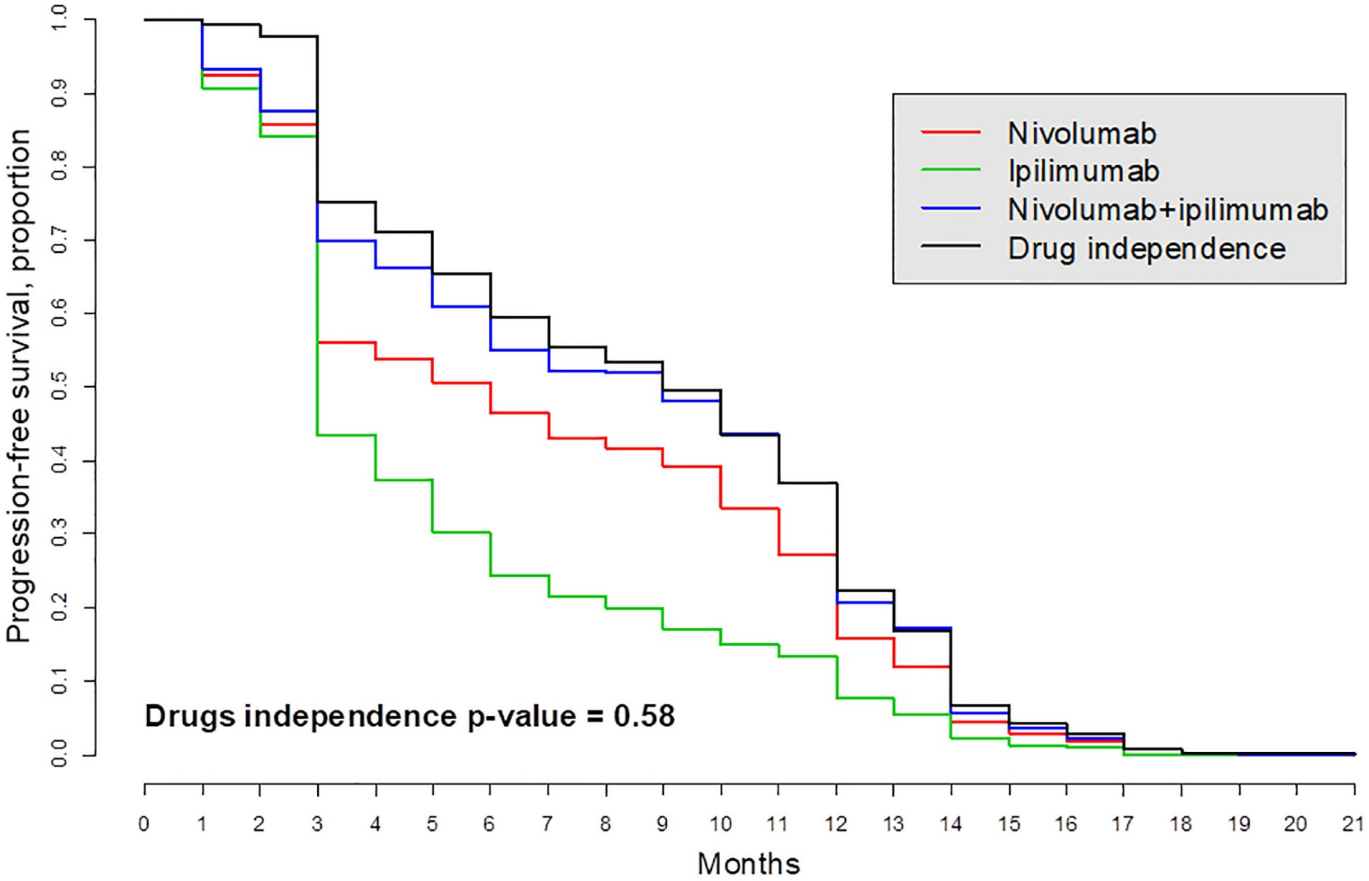
Kaplan-Meier survival curves in “Intention-to-treat population” for treatment metastatic melanoma patients with ipilimumab (drug *A*) and/or nivolumab (drug *B*); see [49]. The survival curve “Drug independence” is computed by formula (12). This curve basically overlaps with the observed survival curve *S*_*AB*_ (blue) which means that the hypothesis of independence cannot be rejected.

**Fig 9:**
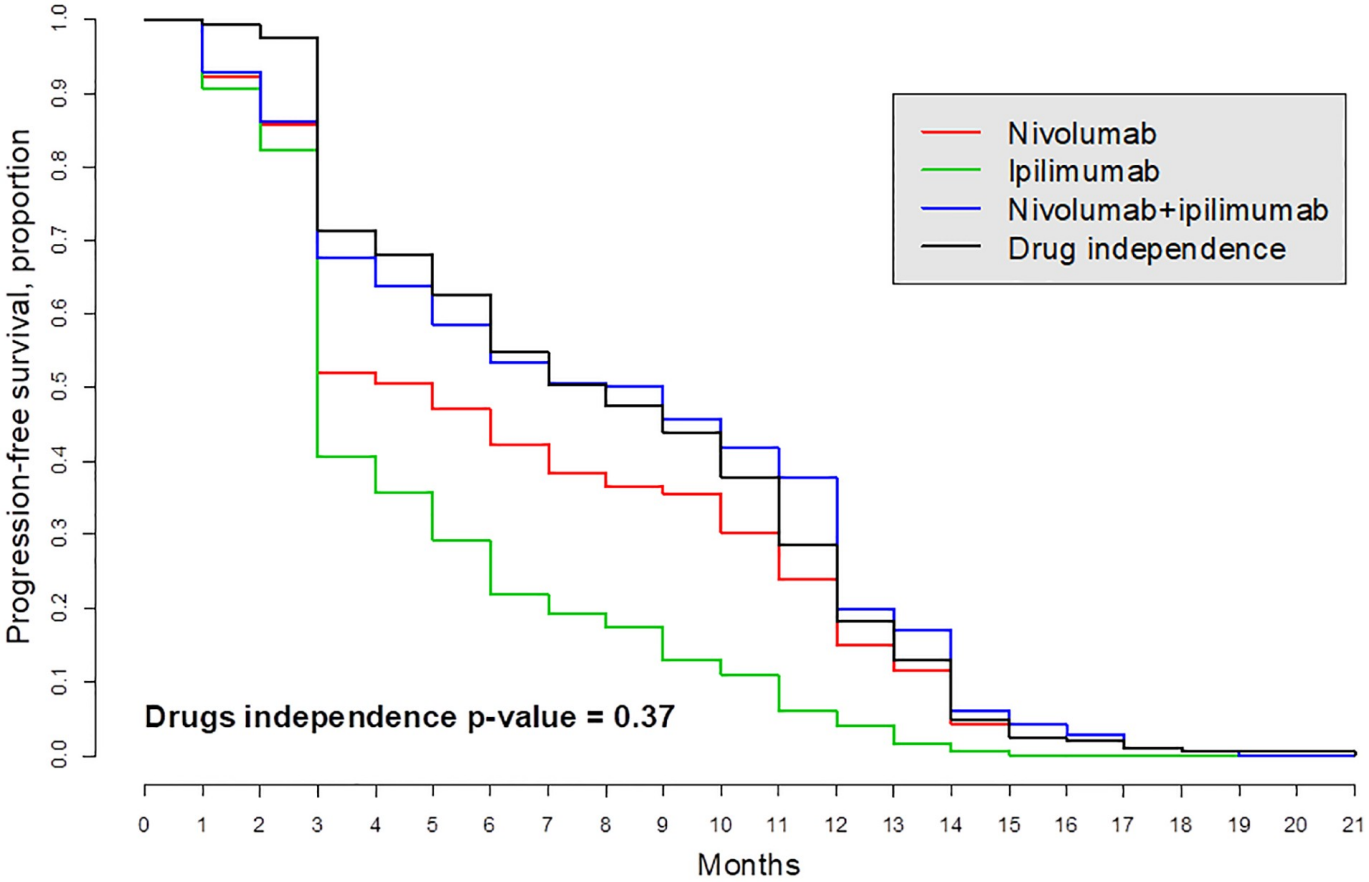
The hypothesis that drugs act independently for patients with PD-L1-negative tumors cannot be rejected because the expected (black) and observed (blue) curves are close; see [49].

We illustrate this approach using a study of the interaction between nivolumab and ipilimumab for the treatment of patients with metastatic melanoma ([49], [50]). Although the authors hint at possible drug synergy, it was not tested. According to our analysis, the drugs acted independently in two groups of patients, “Intent-to-treat population” and “Patients with PD-L1-negative tumors” (Figs 8 and 9). The survival curve denoted as “drugs independence” and computed as the right-hand side of Eq (12) is expected under the assumption that the two drugs acted independently. This curve is very close to the patient-derived survival curve *S*_*AB*_ denoted “Nivolumab + ipilimumab” as evidence that the two drugs act independently. A formal statistical logrank test produced the *p*-values 0.58 and 0.37 (using R package survival) suggesting that the hypothesis that these two drugs acted independently cannot be rejected despite the fact that the survival curve with the drug combination is above the single-drug survival curves.

## Discussion

Bliss definition of drugs independence has a solid probabilistic justification that relies upon the interpretation of the drug effect as the probability that an individual cell or organism dies. Sometimes, Bliss definition of independence is criticized for possibility of claiming synergy when the same drugs are applied. Although theoretically this is a limitation of Bliss definition, fortunately in practice we never split the same drug into two doses. In the end, the endpoint of dose-response experiments is surviving fraction, not our perception of the biological mechanism of synergy. On the other hand, we argue that Loewe additivity definition may claim independence for biologically synergetic drugs. Indeed, imagine two different cancer drugs that act on different set of receptors but equivalent in terms of their individual effect, i.e. they have the same dose-response relationship. In addition, let us assume that cancer cell die if only two set of receptors are damaged, that is, drugs are biologically synergistic. However, if they have the same dose-response relationship, according to Loewe definition, drugs will act independently.

We offer a rigorous and systematic implementation of Bliss independence applied to common design of preclinical (*in vitro* and animal) and clinical (e.g., survival) studies. Statistical evaluation and computation of a *p*- and *d*-value should be a required component when reporting on drug synergy or antagonism because of considerable variation in response to treatment across organisms and replicates. Moreover, the existing shortage of statistical tests for synergy leads to frequent overstatements and false positive findings. Herein, we demonstrated that Bliss independence can be tested using comprehensive statistical models tailored to major biomedical study designs. Unlike prior reports describing statistically testing for drug independence using the traditional *t*-test we developed statistical models that comply with the normal distribution required for correct application of many statistical tests and computation of the *p*-value.

## Supporting information

S1 File R Codes and Appendix. (PDF)

S2 File Raw data on Daphnia experiments with replicates. (CSV)

S3 File Raw data on cancer cell experiments *in vitro* in the presence of control group. (CSV)

S4 File Raw data on tumor growth in mice analyzed by a linear mixed model on the log scale. (CSV)
