# Supplementary material for: Statistical determination of synergy based on Bliss definition of drugs independence
Source: PLoS One. 2019 Nov 25;14(11):e0224137. doi: 10.1371/journal.pone.0224137 (PMC6876842; doi:10.1371/journal.pone.0224137)
Supplement: S1 File — (PDF) [file pone.0224137.s001.pdf]

R Codes and Appendix

Supporting Information
for the manuscript "Statistical determination of synergy based on Bliss definition of drugs independence"
by Eugene Demidenko and Todd W. Miller

The aim of this supplement is to provide complete `R` codes to computations in the paper along with the required data. The public domain R package is freely available at `https://www.R-project.org`. The following *.csv data files are required/provided: `Daphnia.csv`, `ZR75.csv` and `MCF-7_April_2016.csv`. These data files must be stored in the folders specified by each program at the beginning of the code (the folders may be changed).

Treatment interactions with replicates

The Excel file in the .csv format with surviving fraction (SF) data for three treatment groups A, B, and AB must have two columns: the first column is Treatment and the second SF. See file `Daphnia.csv` as an example and the screen snapshot below.

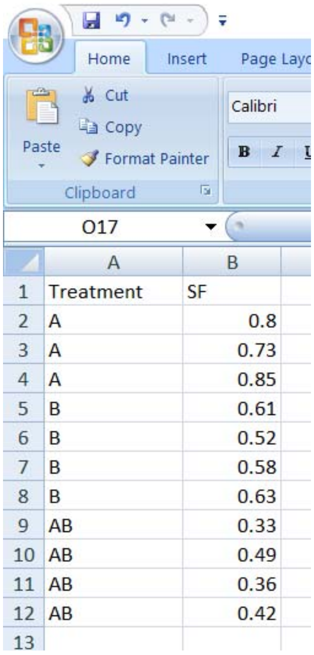

	A	B
1	Treatment	SF
2	A	0.8
3	A	0.73
4	A	0.85
5	B	0.61
6	B	0.52
7	B	0.58
8	B	0.63
9	AB	0.33
10	AB	0.49
11	AB	0.36
12	AB	0.42
13		

The R code for Daphnia example is listed below:

```
daphnia=function(dr="c")
{
#The folder with the data is :\\Projects\\ToddMiller\\Synergy\\Daphnia.csv
da=read.csv(paste(dr,":\\Projects\\ToddMiller\\Synergy\\Daphnia.csv",sep=""))
# names to be displayed
namgr=c("NiCl=1.8","CuSO4=7","NiCl=1.8 & CuSO4=7","Independence")
```

```
tr=da[,1]
A=da[tr=="A",2];B=da[tr=="B",2];D=da[tr=="AB",2] #SFs
n1=length(A);n2=length(B);n3=length(D) #n
lA=log(A);lB=log(B);lD=log(D) #log(SF)
y1=mean(lA);y2=mean(lB);y3=mean(lD)
s1=var(lA)*(n1-1);s2=var(lB)*(n2-1);s3=var(lD)*(n3-1)
sy=s1+s2+s3 # total suam of squares
dft=n1+n2+n3-3;denf=1/n1+1/n2+1/n3 #df
tss=(y1+y2-y3)/sqrt(sum(sy)/dft)/denf    # t-test statistic
pv=2*(1-pt(abs(tss),df=dft)) # p-value for Bliss independence hypothesis
pvP=1-pt(tss,df=dft) # One-sided p-value
print("Testing for Bliss independence:")
print(paste("T-stat=",round(tss,3),", y1+y2-y3=",round(y1+y2-y3,3),
    ", exp(y1+y2-y3)=",round(exp(y1+y2-y3),3),", p-value=",round(pv,3),sep=""))
# Box plot
m=matrix(ncol=3,nrow=4)
m[1:3,1]=lA;m[,2]=lB;m[,3]=lD
indep=rep(0,n1*n2)
k=0
for(i1 in 1:n1)
for(i2 in 1:n2){
    k=k+1
    indep[k]=m[i1,1]+m[i2,2]
}
par(mfrow=c(1,1),mar=c(4,4,1,1))
yl=c(.3,.4,.5,.6,.7,.8,.9) # tickmarks for the original SF
lyl=log(yl) # log tickmarks
boxplot(list(lA,lB,lD,indep),names=namgr,xlab="",ylab="",ylim=range(lyl),axes=F)
mtext(side=1,"Treatment groups",cex=1.25,line=2.75)
mtext(side=2,"Surviving fraction, %",cex=1.25,line=2.5)
axis(side=2,at=lyl,as.character(yl*100))
axis(side=1,at=1:4,namgr)
lines(x=c(3,3,4,4),y=c(log(.55),log(.6),log(.6),log(.55)),lwd=3)
text(3.5,log(.64),paste("P-value =",round(pv,3)),font=2)
yn=log(c(70,50,32,36.5)/100)
text(1:4,yn,paste("n=",c(n1,n2,n3,n1*n2),sep=""),cex=1.25)
}
```

The call `daphnia(dr="d")` produces the folowing output (the data file `Daphnia.csv` must be saved in the folder specified by the `read.csv` command) and Figure 2:

```
> daphnia(dr="d")
[1] "Testing for Bliss independence:"
```

```
[1] "T-stat=1.499, y1+y2-y3=0.156, exp(y1+y2-y3)=1.168, p-value=0.172"
```

Treatment interaction in the presence of control group

The `*.csv` data file has the same format but contains information on the control group (C). The screen snapshot for the data from example on testing the synergy for cancer cells is shown below.

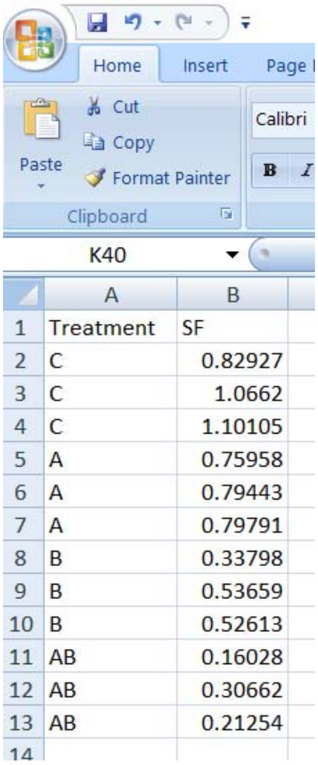

	A	B
1	Treatment	SF
2	C	0.82927
3	C	1.0662
4	C	1.10105
5	A	0.75958
6	A	0.79443
7	A	0.79791
8	B	0.33798
9	B	0.53659
10	B	0.52613
11	AB	0.16028
12	AB	0.30662
13	AB	0.21254
14		

The R code is similar to `daphnia` listed above.

```
bc.control=function(dr="c")
{
#ZR75 cancer line cells
da=read.csv(paste(dr,":\\Projects\\ToddMiller\\Synergy\\ZR75.csv",sep=""))
namgr=c("BYL","GSK","BYL & GSK","Independence")
tr=da[,1]
C=da[tr=="C",2];A=da[tr=="A",2];B=da[tr=="B",2];D=da[tr=="AB",2] #SFs
n0=length(C);n1=length(A);n2=length(B);n3=length(D) #n
lC=log(C);lA=log(A);lB=log(B);lD=log(D)
y0=mean(lC);y1=mean(lA);y2=mean(lB);y3=mean(lD)
s0=var(lC)*(n0-1);s1=var(lA)*(n1-1);s2=var(lB)*(n2-1);s3=var(lD)*(n3-1)
sy=s0+s1+s2+s3
dft=n0+n1+n2+n3-4;denf=1/n0+1/n1+1/n2+1/n3
tss=(y1+y2-y3-y0)/sqrt(sum(sy)/dft)/denf
```

```
pv=2*(1-pt(abs(tss),df=dft))
pvP=1-pt(tss,df=dft) # one-sided p-value
print("Testing for Bliss independence in the presence of the control group:")
print(paste("T-stat=",round(tss,3),", y1+y2-y3-y0=",round(y1+y2-y3,3),"
    , exp(y1+y2-y3-y0)=",round(exp(y1+y2-y3-y0),3),", p-value=",round(pv,3),sep=""))
print(c(sy,dft,denf,tss))
m=cbind(lC,lA,lB,lD)
indep=rep(0,n1*n2*n3)
k=0
for(i1 in 1:n1)
for(i2 in 1:n2)
for(i3 in 1:n0)
{
 k=k+1
 indep[k]=m[i1,2]+m[i2,3]-2*m[i3,1]
}
lCA=rep(0,n1*n0);lCB=rep(0,n2*n0);lCD=rep(0,n3*n0)
k=0
for(i0 in 1:n0)
for(i1 in 1:n1)
{
 k=k+1
 lCA[k]=lA[i1]-lC[i0]
}
k=0
for(i0 in 1:n0)
for(i1 in 1:n1)
{
 k=k+1
 lCB[k]=lB[i1]-lC[i0]
}
k=0
for(i0 in 1:n0)
for(i2 in 1:n2)
{
 k=k+1
 lCB[k]=lB[i2]-lC[i0]
}
k=0
for(i0 in 1:n0)
for(i2 in 1:n3)
{
```

```
 k=k+1
 lCD[k]=lD[i2]-lC[i0]
}
par(mfrow=c(1,1),mar=c(4,4,1,1))

yl=c(.1,.2,.4,.7,1);lyl=log(yl)
boxplot(list(lCA,lCB,lCD,indep),names=namgr,xlab="",ylab="",ylim=range(lyl),axes=F)
mtext(side=1,"Treatment groups",cex=1.25,line=2.75)
mtext(side=2,"Surviving fraction from control, %",cex=1.25,line=2.5)
axis(side=2,at=lyl,as.character(yl*100))
axis(side=1,at=1:4,namgr)
lines(x=c(3,3,4,4),y=c(log(.7),log(.8),log(.8),log(.7)),lwd=3)
text(3.5,log(.88),paste("P-value =",round(pv,3)),font=2)
yn=log(c(60,27,12,18)/100)
text(1:4,yn,paste("n=",c(n1*n0,n2*n0,n3*n0,n0*n1*n2),sep=""),cex=1.25)
}
```

The call for the example using ZR75 treatment data is listed below (it produces Figure 3):

```
> bc.control(dr="d")
[1] "Testing for Bliss independence in the presence of the control group:"
[1] "T-stat=1.691, y1+y2-y3-y0=0.494, exp(y1+y2-y3-y0)=1.653, p-value=0.129"
```

Tumor growth experiments *in vivo*

The tumor growth data in each group is estimated by linear mixed model with random intercept (mice-specific baseline tumor volume) using function `lme` from the library `nlme`, as discussed in detail in Demidenko (2013). The following `R` code plots Figure 6, estimates the rate of slope (beta coefficients) and the one-sided p-value for synergy. Tumor volume is plotted on the log scale with th etickmarks on the original mm^3 scale. Before calling the `tum.growth` function the data file (provided) `MCF-7_April_2016.csv` must be saved in the folder `dr,":\\Projects\\ToddMiller\\` where `dr` is the user defined hard drive (the default is `c`).

```
tum.growth=function(dr="c")
{
library(nlme)
trnam=c("vehicle","EHT1864","Fulvestrant","EHT + Fulvestrant")
da=read.csv(paste(dr,":\\Projects\\ToddMiller\\MCF-7_April_2016.csv",sep=""))
da=da[1:202,]
names(da)=c("week","id","tv","tr")
da$tv=log(da$tv)
par(mfrow=c(2,2),mar=c(4,4,3,1))
rse=matrix(ncol=2,nrow=4)
ytv=c(100,200,400,700,1000);lytv=log(ytv)
```

```
for(itr in 0:3)
{
    dai=da[da$tr==itr,]
    uid=unique(dai$id);nuid=length(uid)
    plot(0:1,0:1,xlim=c(0,50),ylim=range(lytv),type="l",xlab="",ylab="",axes=F)
    mtext(side=1,"Week",line=2.5,cex=1.25);axis(side=1,seq(from=0,to=50,by=10))
    mtext(side=2,"Tumor volume",line=2.5,cex=1.25);axis(side=2,lytv,labels=ytv)
    mtext(side=3,trnam[itr+1],line=.5,cex=1.5)
    for(i in 1:nuid)
    {
        x=dai$week[dai$id==uid[i]]
        y=dai$tv[dai$id==uid[i]]
        y=y[order(x)];x=x[order(x)]
        lines(x,y);points(x,y)
    }
    o=lme(tv~week,random=~1|id,data=dai)
    a=as.vector(o$coefficients$fixed)
    vara=summary(o)$varFix[2,2]
    cat("\n=========================",trnam[itr+1])
    print(summary(o))
    xtt=0:50
    lines(xtt,a[1]+a[2]*xtt,col=2,lwd=3)
    text(10,log(150),paste("Growth rate=",round(a[2]*100,2),"% per week",sep=""),
                            adj=0,cex=1.3)
    if(itr==0) print(sqrt(vara)*log(10))
    if(itr>0)
    {
        Z=abs(a[2]-rse[1,1])/sqrt(vara+rse[1,2])
        pv=round(2*(1-pnorm(Z)),3)
    #    text(1,3.3,paste("P-value difference with control=",pv,sep=""),adj=0,cex=1.3
    }
    rse[itr+1,1]=a[2];rse[itr+1,2]=vara
}
dif=rse[2,1]+rse[3,1]-rse[4,1]-rse[1,1]
vardif=rse[2,2]+rse[3,2]+rse[4,2]+rse[1,2]
Z=dif/sqrt(vardif)
pv=pnorm(-Z)
print(paste("Dif synergy=",dif,", SEdif=",sqrt(vardif),", One-sided p-value=",pv))
text(30,log(800),paste("P-value synergy =",round(pnorm(-Z),4)),cex=1.5,font=2)
}
```

Below is the output of the call:

```
tum.growth(job=4.1,dr="d")
========================== vehicleLinear mixed-effects model fit by REML
 Data:  dai
 AIC BIC logLik
 27.54458 36.11712 -9.772288
Random effects:
 Formula:  ~1 | id
 (Intercept) Residual
StdDev:  0.1885133 0.2378287
Fixed effects:  tv ~week
 Value Std.Error DF t-value p-value
(Intercept) 5.343933 0.10178890 59 52.50015 0
week 0.043158 0.00309586 59 13.94053 0
 Correlation:
 (Intr)
week -0.48
Standardized Within-Group Residuals:
 Min Q1 Med Q3 Max
-2.06278068 -0.58150467 0.09181811 0.72776159 1.77337223
Number of Observations:  65
Number of Groups:  5
[1] 0.007128487
========================== EHT1864Linear mixed-effects model fit by REML
 Data:  dai
 AIC BIC logLik
 15.69589 23.7973 -3.847946
Random effects:
 Formula:  ~1 | id
 (Intercept) Residual
StdDev:  0.1786596 0.2144621
Fixed effects:  tv ~week
 Value Std.Error DF t-value p-value
(Intercept) 5.684737 0.09546868 52 59.54557 0
week 0.033965 0.00305434 52 11.12036 0
 Correlation:
 (Intr)
week -0.453
Standardized Within-Group Residuals:
 Min Q1 Med Q3 Max
-2.5276746 -0.6799118 0.1455981 0.7542422 1.8281999
Number of Observations:  58
Number of Groups:  5
```

```
========================= FulvestrantLinear mixed-effects model fit by REML
 Data:  dai
 AIC BIC logLik
 41.89483 48.44517 -16.94741
Random effects:
 Formula:  ~1 | id
 (Intercept) Residual
StdDev:  0.3312769 0.2887518
Fixed effects:  tv ~week
 Value Std.Error DF t-value p-value
(Intercept) 5.966817 0.18506058 35 32.24251 0e+00
week 0.014315 0.00330086 35 4.33689 1e-04
 Correlation:
 (Intr)
week -0.366
Standardized Within-Group Residuals:
 Min Q1 Med Q3 Max
-1.8447341 -0.4372608 -0.1083321 0.5040163 2.2384441
Number of Observations:  40
Number of Groups:  4
========================= EHT + FulvestrantLinear mixed-effects model fit by REML
 Data:  dai
 AIC BIC logLik
 3.217402 9.661074 2.391299
Random effects:
 Formula:  ~1 | id
 (Intercept) Residual
StdDev:  0.271203 0.1633187
Fixed effects:  tv ~week
 Value Std.Error DF t-value p-value
(Intercept) 6.007220 0.12870692 33 46.67364 0e+00
week -0.009749 0.00229535 33 -4.24723 2e-04
 Correlation:
 (Intr)
week -0.254
Standardized Within-Group Residuals:
 Min Q1 Med Q3 Max
-2.1716649 -0.5598311 -0.1197832 0.6960109 2.2489355
Number of Observations:  39
Number of Groups:  5
[1] "Dif synergy= 0.014871690066532 , SEdif= 0.00592263748022898 ,
                                  One-sided p-value= 0.00601963642388113"
```

Survival curves

The following R function computes survival curve assuming Bliss independence and computes the p-value based on the logrank test using library `survival` (it must be installed before running this program). The call `synergy.surv(job=1)` produces Figure 7 and the call `synergy.surv(job=3)` produces Figure 8. The survival times `ni`, `niip`, and `ip` are taken from Larkin et al. paper (2015).

```
synergy.surv=function(job=1,dr="c")
{
#install.packages("survival")
library(survival)
if(job==1) #Larkin, Fig 1A Intention-to-Treat Population
{
 ni=c(316,292,271,177,170,160,147,136,132,124,106,86,50,38,14,9,6,2,1,1,1,0)
 niip=c(314,293,275,219,208,191,173,164,163,151,137,116,65,54,18,11,7,2,1,0,0,0)
 ip=c(315,285,265,137,118,95,77,68,63,54,47,42,24,17,7,4,3,0,0,0,0,0)
 ti=0:21
 sni=ni/ni[1];sip=ip/ip[1];sniip=niip/niip[1]
 par(mfrow=c(1,1),mar=c(4,4,1,1))
 matplot(ti,cbind(sni,sip,sniip),type="s",lty=1,lwd=3,col=c(2,3,4),
               xlab="",ylab="",axes=F)
 axis(side=1,0:21);axis(side=2,seq(from=0,to=1,by=.1),srt=90)
 sind=1-(1-sni)*(1-sip)
 lines(ti,sind,type="s",lwd=3)
 mtext(side=1,"Months",line=2.75,cex=1.5)
 mtext(side=2,"Progression-free survival, proportion",line=2.5,cex=1.5)
 legend(13,.9,c("Nivolumab","Ipilimumab","Nivolumab+ipilimumab","Drug independence"),
       cex=1.5,lty=1,col=c(2,3,4,1),lwd=3,bg="gray90")
 # Drug independence p-value

 nti=length(niip)
 p.nii=NULL
 for(i in 2:nti)
 if(niip[i]<niip[i-1]) p.nii=c(p.nii,rep(ti[i],niip[i-1]-niip[i]))

 nti=length(sind)
 s.nii=NULL
 for(i in 2:nti)
 if(niip[i]<niip[i-1]) s.nii=c(s.nii,rep(ti[i],round(315*(sind[i-1]-sind[i]))))

 da=as.data.frame(cbind(c(p.nii,s.nii),rep(1,length(p.nii)+length(s.nii)),
          c(rep(1,length(p.nii)),rep(0,length(s.nii)))))
 names(da)=c("dyy","rec","tr")
```

```
 fl.surv <- survfit(Surv(dyy, rec) ~tr, data = da)
 ss=survdiff(Surv(dyy, rec) ~tr, data = da)
 pv=1-pchisq(ss[[5]],df=1)
 text(0,.05,paste("Drugs independence p-value =",round(pv,2)),cex=1.5,adj=0,font=2)

}
if(job==2) #Larkin, Fig 1C Patients with PD-L1-Negative Tumors
{
 ni=c(208,192,178,108,105,98,88,80,76,74,63,50,31,24,9,5,4,2,1,1,1,0)
 niip=c(210,195,181,142,134,123,112,106,105,96,88,79,42,36,13,9,6,2,1,0,0,0)
 ip=c(202,183,166,82,72,59,44,39,35,26,22,12,8,3,1,0,0,0,0,0,0,0)
 #return(cbind(ni,niip,ip))
 ti=0:21
 sni=ni/ni[1];sip=ip/ip[1];sniip=niip/niip[1]
 par(mfrow=c(1,1),mar=c(4,4,1,1))
 matplot(ti,cbind(sni,sip,sniip),type="s",lty=1,lwd=3,col=c(2,3,4),
           xlab="",ylab="",axes=F)
 axis(side=1,0:21);axis(side=2,seq(from=0,to=1,by=.1),srt=90)
 legend(13,.9,c("Nivolumab","Ipilimumab","Nivolumab+ipilimumab","Drug independence"),
       cex=1.5,lty=1,col=c(2,3,4,1),lwd=3,bg="gray90")

 sind=1-(1-sni)*(1-sip)
 lines(ti,sind,type="s",lwd=3)
 mtext(side=1,"Months",line=2.75,cex=1.5)
 mtext(side=2,"Progression-free survival, proportion",line=2.5,cex=1.5)
 # Drug independence p-value

 nti=length(niip)
 p.nii=NULL
 for(i in 2:nti)
 if(niip[i]<niip[i-1]) p.nii=c(p.nii,rep(ti[i],niip[i-1]-niip[i]))

 nti=length(sind)
 s.nii=NULL
 for(i in 2:nti)
 if(niip[i]<niip[i-1]) s.nii=c(s.nii,rep(ti[i],round(315*(sind[i-1]-sind[i]))))

 da=as.data.frame(cbind(c(p.nii,s.nii),rep(1,length(p.nii)+length(s.nii)),
              c(rep(1,length(p.nii)),rep(0,length(s.nii)))))
 names(da)=c("dyy","rec","tr")
 fl.surv <- survfit(Surv(dyy, rec) ~tr, data = da)
 ss=survdiff(Surv(dyy, rec) ~tr, data = da)
```

```
pv=1-pchisq(ss[[5]],df=1)
text(0,.05,paste("Drugs independence p-value =",round(pv,2)),cex=1.5,adj=0,font=2)

}
}
```

Appendix. The two-drug copula and the R code

0.1. Mathematical derivation and properties of the two-drug copula mortality function

Let two drugs A and B have their own singe-agent mortality functions $M_A(\ln d_A)$ and $M_B(\ln d_B)$, where d_A and d_B individual doses. To simplify the notation denote $x = \ln d_A$ and $y = \ln d_B$. The mortality function $M(x)$, as any cdf, must (1) be an increasing function, (2) vanish when x goes to $-\infty$, and (3) approach 1 when x goes to $+\infty$. For example, the popular Hill mortality function $M(d) = (d/EC_{50})^m/(1 + (d/EC_{50})^m)$ can be expressed on the log scale through the logit link, Demidenko (2017). The two-agent mortality function M is built with the help of two probability concepts: the inverse normal cdf Φ^{-1} and the bivariate standard normal density with correlation coefficient ρ. The function M which satisfies the properties formulated in the text, called the copula two-agent mortality function, is created as a double integral

$$M(x, y; \rho) = 1 - \int_{\Phi^{-1}(M_A(x))}^{\infty} \int_{\Phi^{-1}(M_B(y))}^{\infty} \phi(u, v; \rho) du dv \tag{1}$$

where

$$\phi(u, v; \rho) = \frac{1}{2\pi\sqrt{1-\rho^2}} e^{-\frac{1}{2(1-\rho^2)}(u^2-2\rho uv+v^2)}$$

is the bivariate standard normal density with the correlation coefficient ρ.

Property #2 follows from the fact that if $d_B \to 0$ then $y \to -\infty$ and therefore $M_B(y) \to 0$ which implies $\Phi^{-1}(M_B(y)) \to -\infty$. Thus the lower and the upper limits of the inner integral are $-\infty$ and ∞ and integration turns the bivariate normal density into a marginal density,

$$M(x, -\infty; \rho) = 1 - \int_{\Phi^{-1}(M_A(x))}^{\infty} \frac{1}{\sqrt{2\pi}} e^{-\frac{1}{2}u^2} du = 1 - (1 - \Phi(\Phi^{-1}(M_A(x)))) = M_A(x),$$

because $\Phi(x) = \int_{-\infty}^{x} (2\pi)^{-1/2} e^{-\frac{1}{2}u^2} du$ and $\Phi(\Phi^{-1}(M_A(x))) = M_A(x)$. The same proof works for drug B. This means that the copula two-agent mortality function turns into a single-agent mortality function in the absence of the other drug.

Property #3 means that parameter ρ controls the drug interaction. When $\rho = 0$ two drugs act independently which means that function (1) turns into

$$M(x, y; 0) = 1 - (1 - M_A(x))(1 - M_B(y)),$$

the Bliss definition of independence. Indeed,

$$
\begin{aligned}
& M(x,y;0) \\
= \; & 1 - \int_{\Phi^{-1}(M_A(x))}^{\infty} \int_{\Phi^{-1}(M_B(y))}^{\infty} \frac{1}{2\pi} e^{-\frac{1}{2}(u^2+v^2)} dudv \\
= \; & 1 - \left(\int_{\Phi^{-1}(M_A(x))}^{\infty} \frac{1}{\sqrt{2\pi}} e^{-\frac{1}{2}u^2} du \right) \left(\int_{\Phi^{-1}(M_B(y))}^{\infty} \frac{1}{\sqrt{2\pi}} e^{-\frac{1}{2}v^2} dv \right) \\
= \; & 1 - \left(1 - \int_{-\infty}^{\Phi^{-1}(M_A(x))} \frac{1}{\sqrt{2\pi}} e^{-\frac{1}{2}u^2} du \right) \left(1 - \int_{-\infty}^{\Phi^{-1}(M_B(y))} \frac{1}{\sqrt{2\pi}} e^{-\frac{1}{2}v^2} dv \right) \\
= \; & 1 - (1 - M_A(x))(1 - M_B(y)).
\end{aligned}
$$

It is possible to prove that if $\rho > 0$ we have synergy and if $\rho < 0$ we have antagonism as stated in the property #3.

Now we discuss numerical methods for computation our copula two-agent function. To facilitate computation (1) replace the lower finite limit of integration with the upper as follows:

$$
\int_{\Phi^{-1}(M_A(x))}^{\infty} \int_{\Phi^{-1}(M_B(y))}^{\infty} \phi(u,v;\rho) dudv = 1 - \int_{-\infty}^{\Phi^{-1}(M_A(x))} \int_{-\infty}^{\Phi^{-1}(M_B(y))} \phi(u,v;\rho) du
$$
$$
- \int_{-\infty}^{\infty} \int_{-\infty}^{\Phi^{-1}(M_B(y))} \phi(u,v;\rho) du + \int_{-\infty}^{\Phi^{-1}(M_A(x))} \int_{-\infty}^{\Phi^{-1}(M_B(y))} \phi(u,v;\rho) dudv
$$

and therefore (1) takes the form

$$
\int_{-\infty}^{\Phi^{-1}(M_A(x))} dv \int_{-\infty}^{\infty} \phi(u,v;\rho) du + \int_{-\infty}^{\infty} du \int_{-\infty}^{\Phi^{-1}(M_B(y))} \phi(u,v;\rho) dv
$$
$$
- \int_{-\infty}^{\Phi^{-1}(M_A(x))} \int_{-\infty}^{\Phi^{-1}(M_B(y))} \phi(u,v;\rho) dudv.
$$

Finally, we obtain the following more convenient representation of (1):

$$
M(x,y;\rho) = M_A(x) + M_B(y) - \int_{-\infty}^{\Phi^{-1}(M_A(x))} \int_{-\infty}^{\Phi^{-1}(M_B(y))} \phi(u,v;\rho) dudv. \tag{2}
$$

For example, the copula two-agent probit mortality function on the log scale takes the form

$$
M(d_A, d_B) = \Phi(\alpha_A + \beta_A \ln d_A) + \Phi(\alpha_B + \beta_B \ln d_B) - \int_{-\infty}^{\alpha_A + \beta_A \ln d_A} \int_{-\infty}^{\alpha_B + \beta_B \ln d_B} \phi(u,v;\rho) dudv, \tag{3}
$$

where $\alpha_A, \beta_A, \alpha_B, \beta_B$, and ρ are subject to estimation from the dose-response data. A technically similar to (2) but conceptually different probit-based two-drug dose-response model has been suggested earlier in Ashford and Sowden (1970) and Lesaffre and Molenberghs (1991); see also Fedorov

and Leonov (2014). The technical difference is that our integration limit is lower and their integration limit is upper. The conceptual difference is that their model implies probabilistic independence when $\rho = 0$, i.e. the two-drug function collapses to the product of single-drug functions while our model results in Bliss independence.

There are several ways to compute the double integral appeared in (2). First, one can use an `R` function `pmvnorm` from the package `mvtnorm`. Second, the double integral can be reduced to a one-dimensional integral using the formula

$$\int_{-\infty}^{U}\int_{-\infty}^{V}\phi(u,v;\rho) = \int_{-\infty}^{V}\Phi\left(\frac{U-\rho v}{\sqrt{1-\rho^2}}\right)\phi(v)dv$$

where $\phi = \Phi'$ is the density of the standard normal cdf. Using this representation we express (2) as

$$M(x,y;\rho) = M_A(x) + M_B(y) - \int_{-\infty}^{\Phi^{-1}(M_B(y))}\Phi\left(\frac{\Phi^{-1}(M_A(x)) - \rho v}{\sqrt{1-\rho^2}}\right)\phi(v)dv. \quad (4)$$

One-dimensional integration is a built-in function in many computer languages. For example, in R it is `integrate`, and therefore special packages such as `mvtnorm` are not needed if the above formula is used.

Proof of Theorem. The single integral representation formula (4) is used for the proof, where $x = \ln d_A$ and $y = \ln d_B$. (a) Without loss of generality we can assume that $x \geq y$ so that $M_A(x) \geq M_B(y)$ because we assume that $M_A = M_B$. Then we need to prove that $M(x,y;1) = M_A(x)$. When $\rho \to 1$ the denominator of the argument of the Φ function in (4) goes to zero and the numerator converges to $\Phi^{-1}(M_A(x)) - v$. Therefore the argument of Φ converges to either $-\infty$ or $+\infty$ depending on the sign of $\Phi^{-1}(M_A(x)) - v$. Since $M_B(y) \leq M_A(x)$ we have $\Phi^{-1}(M_B(y)) \leq \Phi^{-1}(M_A(x))$. Also we have $v \leq \Phi^{-1}(M_B(y))$ since the right-hand side of this inequality is the upper limit of the integration. Combination of these two inequalities implies that the argument of function Φ converges to $+\infty$ and therefore the integral converges to $\int_{-\infty}^{\Phi^{-1}(M_B(y))}\phi(v)dv = \Phi(\Phi^{-1}(M_B(y))) = M_B(y)$. This means that in the limit $M(x,y;1) = M_A(x) + M_B(y) - M_B(y) = M_A(x)$. (b) When $\rho \to -1$ the argument of Φ converges to $-\infty$ or $+\infty$ depending on the sign of $\Phi^{-1}(M_A(x)) + v$ where $v \leq \Phi^{-1}(M_B(y))$, which implies that $\Phi^{-1}(M_A(x)) + v \leq \Phi^{-1}(M_A(x)) + \Phi^{-1}(M_B(y))$. Consider the case when $M_A(x) + M_B(y) \leq 0.5$. Then both $\Phi^{-1}(M_A(x))$ and $\Phi^{-1}(M_B(y))$ are negative and therefore the argument of Φ in (4) converges to $-\infty$. This means that in the limit $M(x,y;-1) = M_A(x) + M_B(y)$. The proof $M(x,y;-1) = 1$ when $M_A(x) + M_B(y) > 1$ is omitted.

Example: testing lethal effects of insecticides

Below we list the `R` code for estimation of the bivariate copula dose-response model using nonlinear least squares (`nls` function). It requires library `mvtnorm` (install `"mvtnorm"` first). The single dose-response probit models are be estimated using `glm` . See more detail in [2].

```
#install.packages("mvtnorm")
library(mvtnorm)
```

```
bmn2=function(x,y,ab11,ab12,ab21,ab22,ro)
{
    covm=matrix(c(1,ro,ro,1),2,2)
    ar1=ab11+ab12*x;ar2=ab21+ab22*y
    pnorm(ar1)+pnorm(ar2)-pmvnorm(upper=c(ar1,ar2),sigma=covm)
}
bmn2.rc=function(x,y,ab11,ab12,ab21,ab22,ro)
{
    covm=matrix(c(1,ro,ro,1),2,2)
    ar1=ab11+ab12*x;ar2=ab21+ab22*y

    n=length(ar1)
    pred=rep(0,n)
    for(i in 1:n)
        pred[i]=pnorm(ar1[i])+pnorm(ar2[i])-pmvnorm(upper=c(ar1[i],ar2[i]),sigma=covm
    return(pred)
# 1:5 mixture Finney data
    #Rotenon (R)
    dR1=c(.1,.15,.2,.25,.35);KR1=c(.24,.44,.63,.81,.9);LdR1=log(dR1)
    dR2=c(.1,.15,.2,.25,.35);KR2=c(.28,.51,.72,.82,.89);LdR2=log(dR2)
    xR=c(LdR1,LdR2);yR=c(KR1,KR2)
    #o=glm(yR~xR,family=binomial(probit));aR=as.vector(coef(o)) #estimation of a
single dose-response probit model
    #plot(aR[1]+aR[2]*xR,log(y/(1-y)))
    #print(summary(o))

    #Pyrethrins (P)
    dP1=c(.5,.75,1,1.5,2);KP1=c(.2,.35,.53,.8,.88);LdP1=log(dP1)
    dP2=c(.5,.75,1,1.5,2);KP2=c(.23,.44,.55,.72,.9);LdP2=log(dP2)

    xP=c(LdP1,LdP2);yP=c(KP1,KP2)
    #o=glm(yP~xP,family=binomial(probit));aP=as.vector(coef(o))
#    plot(aP[1]+aP[2]*xP,log(y/(1-y)))
    #print(summary(o))

    # 1:5 mixture
    d15=c(.3,.45,.6,.875,1.175);Kd15=c(.27,.53,.64,.82,.93)
    Ld15R=log(d15/6);Ld15P=log(d15*5/6)
    x1C=c(xR,rep(mINF,10),Ld15R);x2C=c(rep(mINF,10),xP,Ld15P)
    yC=c(yR,yP,Kd15)
    oC=nls(yC~bmn2.rc(x=x1C,y=x2C,ab11,ab12,ab21,ab22,ro),
                start=c(ab11=3,ab12=1.5,ab21=.15,ab22=1.5,ro=-.6))
```

```
# ab11=interc_rot,ab12=slope_rot,ab21=interc_pyr,ab22=slope_pyr
    aC=coef(oC)
    print(summary(oC))
```

References

Demidenko, E. (2103). Mixed Models: Theory and Applications in R. Hoboken: Wiley.

Demidenko, E., Glaholt, S.P., Kyker-Snowman, E., Shaw, J.R., Chen, C.Y. (2017). Single toxin dose-response models revisited. Toxicology and Applied Pharmacology 314, 12–23.

Larkin, J., Chiarion-Sileni, V., Gonzalez, R.,et al. (2015). Combined nivolumab and ipilimumab or monotherapy in untreated melanoma. New England Journal of Medicine 373, 23–34.

R Core Team (2017). R: A language and environment for statistical computing. R Foundation for Statistical Computing, Vienna, Austria. URL https://www.R-project.org/.
